# Fluorescence Imaging as a Tool in Preclinical Evaluation of Polymer-Based Nano-DDS Systems Intended for Cancer Treatment

**DOI:** 10.3390/pharmaceutics11090471

**Published:** 2019-09-12

**Authors:** Tomáš Etrych, Olga Janoušková, Petr Chytil

**Affiliations:** Institute of Macromolecular Chemistry, Czech Academy of Sciences, Heyrovského nám. 2, 162 06 Prague 6, Czech Republic

**Keywords:** fluorescence imaging, drug delivery, polymers, noninvasive imaging, theranostics

## Abstract

Targeted drug delivery using nano-sized carrier systems with targeting functions to malignant and inflammatory tissue and tailored controlled drug release inside targeted tissues or cells has been and is still intensively studied. A detailed understanding of the correlation between the pharmacokinetic properties and structure of the nano-sized carrier is crucial for the successful transition of targeted drug delivery nanomedicines into clinical practice. In preclinical research in particular, fluorescence imaging has become one of the most commonly used powerful imaging tools. Increasing numbers of suitable fluorescent dyes that are excitable in the visible to near-infrared (NIR) wavelengths of the spectrum and the non-invasive nature of the method have significantly expanded the applicability of fluorescence imaging. This chapter summarizes non-invasive fluorescence-based imaging methods and discusses their potential advantages and limitations in the field of drug delivery, especially in anticancer therapy. This chapter focuses on fluorescent imaging from the cellular level up to the highly sophisticated three-dimensional imaging modality at a systemic level. Moreover, we describe the possibility for simultaneous treatment and imaging using fluorescence theranostics and the combination of different imaging techniques, e.g., fluorescence imaging with computed tomography.

## 1. Introduction

Hundreds of new drug delivery systems (DDS) have been described within the last decade [1]. To determine the potential of these DDS, various in vitro and in vivo techniques have been applied and studied in detail. Among other methods to evaluate the cytotoxicity, uptake, and binding efficacy to selected cells or the in vivo therapeutic potential to target tissues and cells, visualization techniques play a major and irreplaceable role in determining the therapeutic capacity of novel DDS. In general, optical imaging (OI) is the most universal and commonly used visualization technique in basic research, development, and preclinical studies [2]. In contrast to other imaging techniques, several advantages of this technique are undisputable. In particular, OI greatly reduces “patient” exposure to damaging radiation, i.e., in contrast to radio imaging. OI uses non-ionizing radiation, which is usually composed of visible, ultraviolet, and infrared light-generated images via the excitation of electrons, in the absence of the injury-associated ionizing radiation that is frequently used in other imaging techniques [3]. Thus, OI is an appropriate method for lengthy or repeated procedures over time to monitor disease or treatment progression because it presents a low risk for patients and is typically faster in scanning of the object. From the macroscopic imaging perspective, OI can serve as a powerful tool for research and clinical practice, from high-throughput screening in biology and genetics to non-invasive imaging of functional contrast agents in the desired, intact tissues [4].

In contrast to other imaging methods, OI technologies are usually convenient in term of application, e.g., are modular in design, are portable and compact, and should be arranged at the laboratory bench. In biomedical research, OI can take advantage of a wide range of interactions between the light and the tissue, as well as corresponding photo-physical and photo-chemical mechanisms and processes at the molecular level (e.g., multiphoton absorption, second-harmonic generation, fluorescence, and luminescence). Finally, OI technologies provide a highly multi-functional platform for biomedical research that can be used from molecular to systemic levels and supply a crucial understanding of several phenomena in medicine and research [5]. In cancer research in particular, OI is primarily used, in addition to in vitro applications, for the localization of tumors and metastases, the monitoring of disease progression or regression and the determination of the pharmacokinetics of newly developed DDS.

In the last several decades, nano-sized DDS have been studied intensively as suitable novel nanomedicines for the treatment of various diseases, including neoplastic solid tumors and disseminated hematological malignancies [6]. These nano-sized DDS are generally macromolecules, ranging from 1 to 100 nm in at least one dimension, which deliver the drug either attached or loaded into the carrier structure. Moreover, they are tailored for targeted delivery and/or controlled release of the drug. The nano-sized DDS significantly increase the solubility of drugs, reduce the systemic toxicity of the carried drugs, prolong the circulation and accumulate in the target tissue, thereby highly favoring improved drug pharmacokinetics [1]. The controlled drug release from DDS is achieved predominantly by pairing the carrier system with a stimulus-activated release mechanism. Either an internal specific stimulus, e.g., the pH gradient or hypoxia-driven reductive gradient between the blood and the tumor environment, or external sources, e.g., applied hyperthermia, a magnetic field, and light, activate release of the attached or loaded drug [7]. In general, the use of DDS enable more efficient delivery of drugs to specific pathological sites, the target tissues, and release of the drug into the diseased cells or in their direct surroundings, thus reaching and maintaining the sufficient concentration to eliminate all diseased cells. To date, a large number of studies have been published demonstrating the advantages of DDS over freely soluble drugs, at basic research, preclinical and clinical levels [1,8,9,10]. Upon intravenous (*i.v.*) or intraperitoneal (*i.p.*) administration, the prolonged circulation times and increased hydrodynamic diameter of nanomedicines lead to passive accumulation in solid tumors, metastasis or at sites of inflammation via the enhanced permeability and retention (EPR) effect, [11,12] a vascular phenomenon that involves the extravasation and retention of macromolecules. The EPR effect is driven by incorrect tumor blood vessels and impaired lymphatic drainage due to rapid unorganized solid tumor and metastatic growth. Simultaneously, the nano-sized dimensions of the DDS prevent them from accumulating in healthy organs and tissues, as observed for carrier systems with dimensions in the upper-nano and micro ranges [13]. Finally, an increase in drug levels at target sites and reduction of drug concentrations in potentially endangered healthy tissues support the use of nano-sized DDS to achieve significant improvements in the balance between therapeutic efficacy and unwanted and treatment-limiting side effects [14,15].

A detailed understanding of the above-mentioned DDS-mediated drug targeting systems is highly relevant for the translation of novel DDS from basic research and preclinical development into clinic applications [16]. Quantitative assessments of the biodistribution, target site localization, and accumulation in healthy organs and tissues are key points and tasks in DDS development. Determination of the pharmacokinetics of DDS can be conducted after *i.v*. administration either invasively, i.e., by collecting blood, tumor or tissue samples, or non-invasively, i.e., by using various imaging techniques. Several non-invasive imaging methods are commonly used for monitoring the DDS biodistribution and target site accumulation, e.g., positron emission tomography (PET), single photon emission computed tomography (SPECT), magnetic resonance imaging (MRI), and fluorescence optical imaging (FI) [5,17,18,19]. Nevertheless, the use of radio-based imaging techniques, SPECT and PET, generally enable visualization of the biodistribution for a few hours to a few days due to the short half-life of the used tracers [20,21,22]. Conversely, FI is the most frequently used imaging modality for the non-invasive long-term visualization of DDS in vivo. Based on the time and cost effectiveness and its capability for long-term, high-throughput and simple analyses, FI has become widely used in recent years and is about to surpass nuclear medicine and MR-based imaging techniques in preclinical development in the drug delivery field.

From the physical perspective, in vitro and in vivo FI are based on the illumination of a target tissue or cells with an ultraviolet to infrared light source of a specific wavelength that excites the fluorophores used in the experiment. The excitation light, photons, penetrate through a number of tissue layers in vivo to the fluorophores and thus are partially reflected, scattered, and absorbed by various types of molecules and tissue components [4]. The interaction of the photons with the fluorophores leads to excitation of the fluorophores. The return to the basal energetic state is linked to photon emission at specific wavelengths from the fluorophore. The majority of FI methods and systems currently applied in basic research and preclinical development are based on a planar epi-illumination method known as 2D-fluorescence reflectance imaging (FRI) [23,24]. Briefly, general photographic techniques are used in fluorescence mode to non-invasively capture surface and subsurface fluorescence activity from cells, spheroids, and entire animals. The emitted light is collected on the same side that is exposed to the light source with a highly sensitive charge-coupled device (CCD) chip camera using appropriate filters. Due to the involvement of the easy epi-illumination method, FRI methods are simple from an instrumental perspective, simple to operate and provide high-throughput data. Based on the described advantages, they have gained wide popularity and have assisted in significant advancements in the field of fluorescent molecular imaging [25,26,27]. Nevertheless, significant drawbacks of the FRI method are related to basic limitations in depth resolution, i.e., the method is unable to resolve nonlinear dependencies of the propagated signals detected from the surrounding tissue [28]. Although the basic fluorescence intensity is linearly dependent on the fluorochrome concentration, it has a strong nonlinear dependence on the optical properties of the tissue through which the light is passing, and the tissue depth. Two similar tumors with identical fluorophore contents at two different tissue depths will have significantly different fluorescence intensities in FRI. Similarly, two tumors at the same depth with the same fluorochrome concentration, but different vasculatures, will report different intensities. The more vascularized the tumor (i.e., higher hemoglobin concentrations) will yield a markedly lower fluorescence intensity because of the increased photon absorption by the vascular compartment.

**Figure 1:**
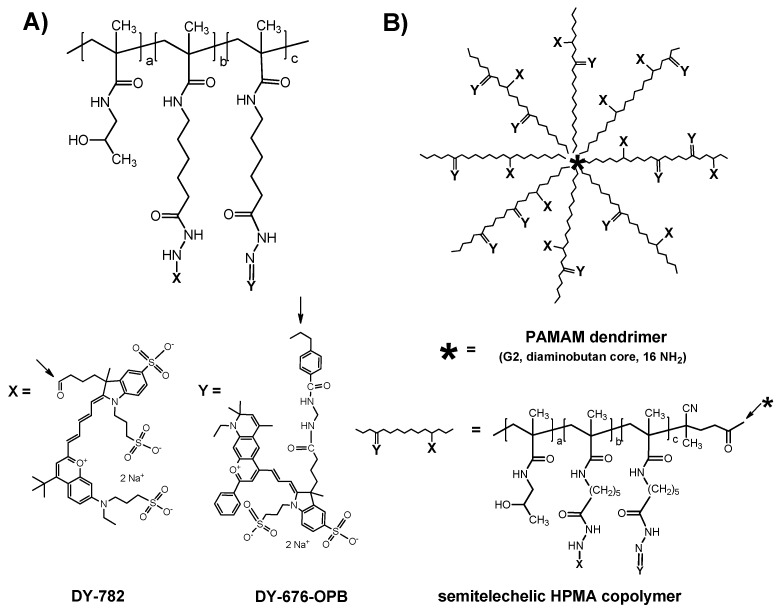
Schematic structure of dual-labeled polymer carriers with Dyomics-782 bound via a non-degradable hydrazide bond and Dyomics-676 bound via a pH-sensitive hydrazone bond—(**A**) linear *N*-(2-hydroxypropyl)methacrylamide (HPMA)-based polymer and (**B**) star HPMA-based polymer. Reprinted with permission from [31], Copyright [2012], American Chemical Society.

Although the quantification and following interpretation of the in vivo FRI results are non-trivial, the advantages of FRI are beneficial when compared with those of other non-invasive imaging techniques (e.g., radionuclide-based approaches or MRI). As described previously, the main advantages of FRI lies in its relatively easy setup (e.g., no radioactive labels), potential for long-term observation (up to several months), and simultaneous use of two or more fluorescent probes [29,30]. The utilization of two different fluorescent dyes in combination with multispectral FRI can serve as a highly innovative platform for the simultaneous visualization of polymeric carrier pharmacokinetics and model drug release in solid tumors (see their chemical structures in Figure 1) [31,32]. It has been shown that the simultaneous collection of information on the biodistribution for both the model drug and the polymer drug carriers can be obtained by FRI (see Figure 5d in the whole body imaging section), not only in the tumor tissue but also in the kidney during elimination of both the polymer carrier and model drug.

Moreover, other in vivo factors have recently been investigated in tumor-bearing mice using dual fluorescently labeled polymer systems, i.e., mainly the impact of the structure of the biodegradable spacer on degradability in vivo, the release rates of a fluorescent model drug conjugated to *N*-(2-hydroxypropyl)methacrylamide (HPMA) copolymers with various pH-sensitive, reductively sensitive and enzymatically biodegradable spacers and the biodistribution of the model drug and polymer carrier itself.

Nevertheless, the fluorescent probes and methods used for their application can greatly affect various processes, including the ligand specificity, intracellular targeting efficacy, concentration ranges for target detection, optimal resolution, and sensitivity. The increasing availability of fluorescent dyes [33,34,35], smart fluorophores [36,37], quantum dots [34,38], metallic nanoparticles [39], composite nanoshells [36], or fluorescent proteins [40,41,42] has facilitated the development and applicability of FI in recent years. From the polymer-based DDS point of view the best candidates are the fluorescent dyes, as they are small in size with respect to the polymer DDS systems. This means that they do not significantly affect the behavior of the DDS itself. Generally, the minimum possible amount of fluorophores is used for DDS labeling to fulfill the crucial condition defined above. Interestingly, some anticancer drugs are known to have fluorescent properties, e.g., doxorubicin and ellipticine, and can serve as extrinsic fluorescent dyes [43]. Thus, partially real-time drug distribution factors, such as localization, tumor accumulation can be directly studied using the drug combining the therapeutic and diagnostic functions. Unfortunately, the excitation and emission wavelengths of these fluorescent drugs limit their applicability in vivo, as the penetration of the light in range 450–560 nm in tissues is highly limited to a few mm. Indeed, ex vivo OI could be performed by these fluorescent drugs more precisely [43].

Generally, the biological evaluation of DDS can be divided into three fields: (I) in vitro studies, (II) preclinical in vivo animal models, and (III) clinical trials in humans. The application of FI will be described in more detail for the first two fields. The text herein will focus on the utilization of FI for in vitro cell studies and in vivo pharmacokinetic studies of advanced DDS with treatment and diagnostic capabilities. Finally, future prospects for advanced FI techniques in DDS research will be discussed.

## 2. Imaging at the Cellular Level

Optical imaging at the molecular level is a rapidly developing technology that allows non-invasive monitoring of biological processes during physiological or pathological conditions at cellular and subcellular level in cells and tissues. Fluorescence, together with bioluminescence, belongs to the most important optical imaging modalities, representing methods for the simple and direct observation of specific molecular targets or biological pathways. The current preclinical development of these techniques is based on the increasing availability of fluorescent dyes, proteins, and probes in combination with nanotechnology, within a highly evolving field of interdisciplinary research that enables the fabrication of materials with nanoscale dimensions. Furthermore, the development of novel multi-functional agents based on nanoparticles enables the conjugation of various biologically active molecules, e.g., targeting moieties and therapeutic agents, with imaging probes. The combination of multi-functional agents offers the noninvasive detection of various genes or proteins, protein–protein interactions, and cellular processes that are closely related to pathogenesis, e.g., protease activity, apoptosis, autophagy, or necrosis [38,39,44]. In vitro studies enable the unique opportunity to study in detail the molecular processes of diseases and, likewise, the treatment efficacy. To design and finally produce a viable diagnostic or/and therapeutic tool, the initial steps require the (a) identification of specific markers to be targeted for diagnostic or therapeutic purposes, together with biomarkers of the normal and disease state of the tissue; (b) evaluation of the drug or diagnostic agent targeting /efficacy of action at the cellular level; (c) pre-evaluation of the appropriate carriers of drugs or diagnostic agents, which must also be evaluated for their in vivo delivery efficacy.

For the initial screening, a large number of immortalized cancer cell lines is available commercially or within the scientific community and can be easily used by researchers. These cell lines serve as a basic, but highly valuable, platform to investigate specific molecular features of cancer biology and to explore the potential efficacy of DDS, the anticancer drugs themselves or diagnostic agents. Nowadays, utilization of tumor spheres as in vitro tumor models offers unique possibility to evaluate the biological behavior of various diagnostic and therapeutic agents. The tumor sphere models enable closer view of agents’ penetration, intracellular localization, or toxicity than adhesive cell line models [45].

Within the FI experiment, one can utilize sophisticated fluorescent probes that are tailored as targets for a specific receptor or enzyme. These probes are essentially fluorochromes that are bound to a ligand that is specific for a certain target, such as a monoclonal antibody [46,47], antibody fragment [48,49], peptide [50,51] or aptamer [52]. The probes can be permanently active or activatable by changes in conformation or chemical structure. Activatable probes are usually designed as quenched fluorochromes that are originally inactive fluorochromes and are activated during the experiment. In general, the fluorochromes are either self-quenched, or quenched by a quencher that is positioned, for example, via an enzyme-specific peptide sequence to a fluorochrome [53,54,55]. Thus, the fluorochrome emits light upon excitation only after enzymatic cleavage or another activation procedure. Such probes are known as beacons or smart probes. Many fluorochromes are applicable for in vitro studies because in such analyses one can operate within broad spectrum of optical wavelengths.

In addition, the introduction of fluorescent proteins (FPs) by introducing the transgene into cells is another option to efficiently study biological processes using FI. After expression, the FP serves as an intrinsic fluorescent reporter probe [41,56]. Stably modified cells expressing FP under the control of promotor represent a highly sophisticated tool for studying gene regulation. The fusion of FP-encoding genes with genes of interest enables the possibility of localizing and quantifying specific proteins in vitro and in vivo, especially in cancer research [42,57,58,59]. This approach has become a powerful tool in preclinical research.

In general, data from in vivo experiments afford the highest relevance for preclinical research and further medical applications. Nevertheless, the in vitro evaluation of drug trafficking and accumulation in intracellular targets (organelle or compartment) at adequate concentrations provides indisputable preliminary information for the following therapeutic purposes. At the cellular level, the nano-device size, shape, and charge, and even the flexibility [60,61,62] and morphology [63,64], determine the rate of internalization, the intracellular localization, the anticancer drug release profile, and the degradation of DDS. The efficacy of active targeting using monoclonal antibodies, their fragments, selected oligopeptides or saccharides could be readily evaluated to confirm the primary concept and design of targeted DDS [49,65,66]. Moreover, most DDS based on polymer carriers, micelles, nanoparticles, or liposomes can be easily labeled by different fluorochromes; thus, various biological characteristics and processes can be studied in detail by fluorescence microscopy [31,67,68].

FI using in vitro cell-based models, e.g., cell lines or spheroids, is a powerful tool for studying the characteristics of novel DDS at the cellular level and for comparing their properties, e.g., uptake, kinetics of cellular transport, efflux rates, and drug localization, with the parent free drug or other types of DDS [69,70]. It is generally accepted that the parent free low-molecular-weight drugs are internalized into cells by diffusion more rapidly than DDS, which must enter cells via the slower process of fluid-phase endocytosis or after coupling to a specific receptor by receptor-mediated endocytosis. In vitro, FI can recognize processes by which the proposed DDS enter the cells and the speed of the processes. It also provides information concerning whether the drug or drug model can be released within cells, where the release occurs and what is the rate of drug release, as well as how the drug is re-distributed to the organelles [70,71,72,73]. Indeed, evaluations of anticancer drug effects at the cellular level can be effectively combined with the determination of specific processes, e.g., cell-death pathways, proteolytic processes, protein expression, and co-localizations with organelles and specific proteins [74].

**Figure 2:**
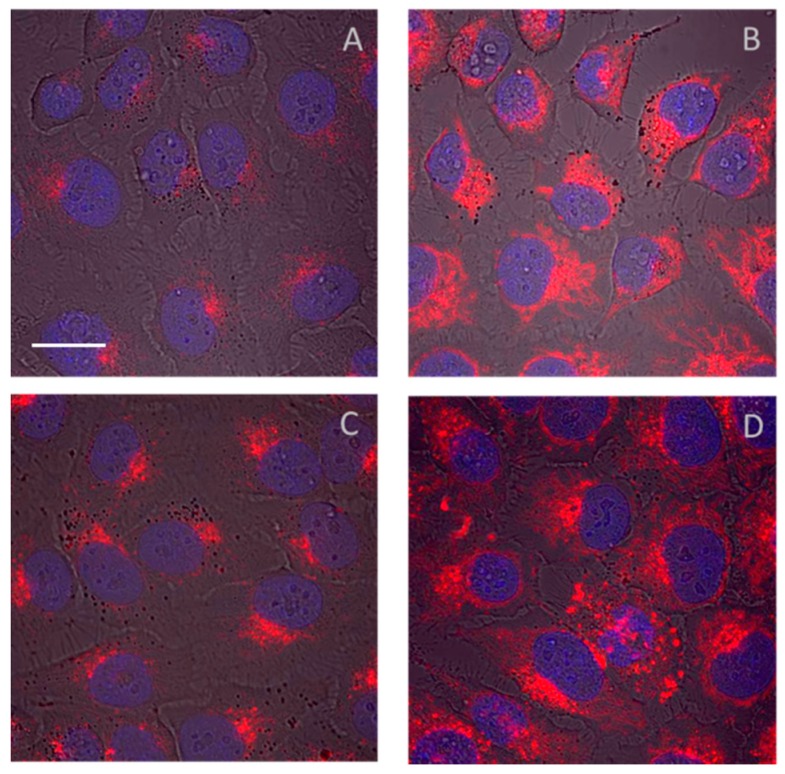
Intracellular localization of pHPMA polymer conjugate labeled with Dyomix 630 (**A**), Cy5 (**B**), ATTO647 (**C**), and Cyanine 5.5 (**D**). The HeLa cells were incubated for 24 h with the labeled polymers. The amount of fluorescently labeled polymer conjugates added to the cell suspensions was normalized to the dye content (1 μg/mL). The fluorescently labeled polymers (red color) were excited at 647 nm, and the emitted light was detected using a 640–740 nm filter. Nuclei were visualized by labeling with Hoechst 33342 dye excited at 405 nm, and emitted light was detected using a 425–500 nm filter, scale bar 20 µm.

In addition to the direct effects of the drug on cells, the fate of the drug carriers after active drug release within the cell compartments can be determined by FI in vitro [70,75,76]. This process can improve the future design of suitable DDS for highly effective delivery of diagnostic agents or drugs within the target tissue. A few anticancer drugs are known to have fluorescent properties, e.g., doxorubicin (Dox) and ellipticine, and can serve as extrinsic fluorescent dyes. Thus, real-time drug distribution factors, such as localization, accumulation, and time and dose-dependent cell death, can be directly studied using the drug combining the therapeutic and diagnostic functions. Moreover, the simultaneous observation of both the drug and DDS carrier can be achieved using two different fluorescent dyes: a drug model dye or fluorescent drug (attached via biodegradable spacers) and a polymer label (attached via non-degradable covalent bonds) [76]. Unfortunately, the fluorescent dyes used for labeling the drug carriers can greatly affect the interaction of the DDS with cell membranes or their uptake into cells. Commercially available fluorophores are usually highly charged or even amphiphilic molecules, and thus their interaction with biological membranes and molecules can occur. Similarly, the endocytic pathways of the studied DDS can be significantly varied and affected, thus leading to misunderstandings and misleading data. Figure 2 shows a comparison of the same DDS based on the HPMA copolymers labeled with four different fluorescent dyes: ATTO-647, Dyomix 630, Cyanine 5, or Cyanine 5.5 dye. In this case, the fluorescent signal was evaluated using the laser scanning confocal microscope (LSCM) Olympus IX83 with FV10-ASW software (version 04.02.03.06).

The confocal microscopy images unequivocally revealed the dye structure-dependent internalization in HeLa cells, which was highly elevated in the case of Cyanine 5.5 and Cyanine 5 dye labeling. Thus, the appropriate selection of fluorescent dye is a key point for in vitro preclinical research. Moreover, the internalization process could also be influenced by the type of cancer cells, and thus the use of more than one cell line is necessary. Due to the potential impact of the dye structure, the biochemical changes (i.e., cell viability, apoptotic changes, and expression of specific proteins, among others) caused by DDS should be evaluated using unlabeled DDS. In the case of fluorescently labeled DDS, the use of appropriate controls, i.e., fluorescently labeled carriers without any drug, is optimal.

**Figure 3:**
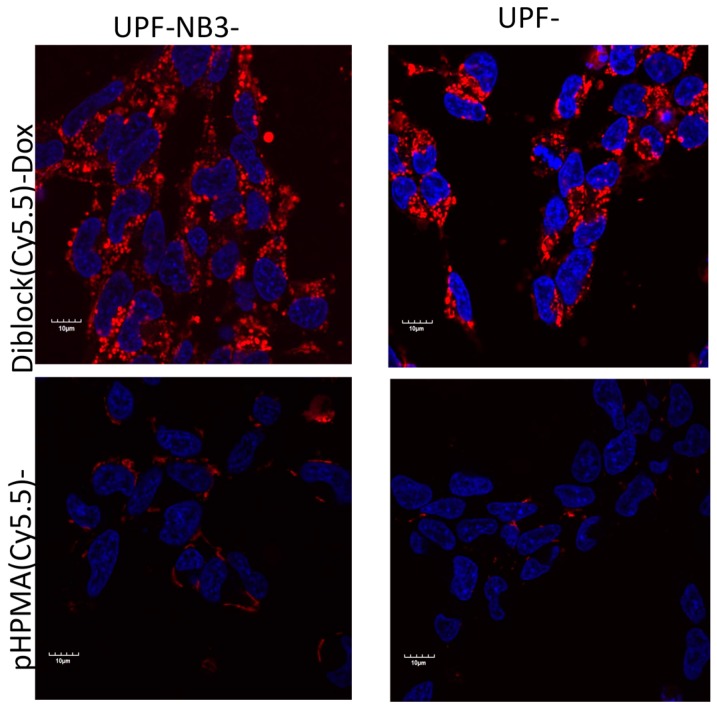
Uptake of polymer conjugates labeled with the fluorescent dye Cyanine 5.5 (shown in red) after incubation of the parental human neuroblastoma UPF-NB3 or resistant UPF-NB3-Dox cell line for 4 h with fluorescently labeled polymer conjugates (micellar PPO-p(HPMA)-(Cy5.5)-Dox (upper row) and linear pHPMA(Cy5.5)-Dox (bottom row)) at 37 °C and 5% CO_2_. The nuclei are stained with Hoechst 33342 (shown in blue). Reprinted with permission from [78], Copyright [2017], Elsevier.

Laser scanning confocal microscopy (LSCM) is a commonly used technique in preclinical research to obtain high-resolution optical images at controllable depths. It is suitable for the in vitro study of active targeting, internalization rates, intracellular distributions, and co-localization of drug and DDS, among others. As an example, we present the LSCM evaluation of the polymer conjugate intracellular accumulation, which was conducted in a study focusing on overcoming multi-drug resistance (MDR) based on ATP Binding Cassette (ABC) transporters using amphiphilic diblock polymer nanotherapeutics bearing Dox (attached by a biodegradable spacer to the polymer carrier) in Dox-resistant human neuroblastoma cell lines overexpressing ABC transporters. In general, ABC transporters are efflux pumps that actively pump out drugs or, more generally, xenobiotic compounds. Their overexpression in MDR cells leads to insufficient, potentially sub-therapeutic intracellular drug concentrations. Recently, micelle-forming diblock copolymers composed of poly(propylene oxide) (PPO) block a potential MDR inhibitor linked to the hydrophilic HPMA copolymer block (pHPMA) bearing Dox bound to a pH-sensitive hydrazone bond were evaluated using FI in vitro in various MDR-cell lines and sensitive cell lines. Figure 3 shows the significantly different accumulation of polymer carriers in cells, both resistant and sensitive neuroblastoma cell lines UPF-NB3, when compared with the behavior of the control pHPMA polymer carrier incubated in the same cells. More effective accumulation was probably caused by an interaction of the amphiphilic copolymer with cell membranes, leading to enhanced cell internalization [77].

Quenching of fluorescent intensity can be used for observation of DDS behavior in cells. The rate of enzymatic cleavage of spacer between drug and carrier [78,79] or multiblock polymer carriers [80] can be determined using Förster resonance energy transfer (FRET) in vitro. The loss of the FRET signal indicates that the donor fluorophore is not close enough to the acceptor fluorophore, e.g., because of the drug or dye release from the carrier [79]. Nevertheless, not all FRET pairs are suitable for in vivo evaluation, only those which operate in the NIR part of the spectra [81].

Advanced FI methods, such as fluorescence-lifetime imaging microscopy (FLIM), can also be applied at the cellular level. FLIM produces images based on the differences in the exponential decay rate of the fluorescent sample. FLIM is based on changes to the fluorophore lifetime due to factors in the local environment, e.g., pH. This method is highly valuable for the evaluation of drug release profiles and localization and the trafficking of DDS in vitro. FLIM can also be used to simultaneously study DDS trafficking and drug colocalization in specific organelles [82,83].

To sum up this section, the principles of FI in vitro evaluation of DDS at the cellular level were outlined, and the importance of this technique for preclinical research in the field of anticancer drug delivery was discussed. Studies of the intracellular fate of DDS in cancer cell lines of different origins could eliminate non-efficient and non-suitable DDS before the initiation of more expensive and more demanding in vivo evaluations.

## 3. In Vivo Imaging

In principle whole body FRI is from an instrumental and processing perspective similar to FI at the cellular level. Nevertheless, the complexity of the living animal system introduces some limitations but affords more relevant insight into the processes in the organism. In addition to adsorption and scattering, autofluorescence (AF) is another important factor. The degree of absorption and AF depends on the range of the excitation wavelength of the body compartments [84,85]. Namely, the absorption of hemoglobin and myoglobin and the AF of collagen, elastin, and tryptophan, among others, prevent reasonable and sensitive FRI in the ultraviolet to red spectral range (200 to 650 nm). In addition, skin pigment melanin, fur, and the size of the laboratory animals are limiting factors. Therefore, the ideal wavelength range comprises the NIR spectral region (650 to 900 nm) in which the tissue absorption and AF are suppressed [84,85]. The choice of suitable laboratory animals for studies with human model tumors is reduced to athymic nude mice due to their short lifetime, fast reproducibility, relatively low cost of handling, housing, and breeding and the opportunity to use human xenografts. Athymic nude mice have no functional thymus and, therefore, no circulating T-cells. The resultant deficient specific immune response prevents the rejection of transplanted human cells and tissues. However, the main drawback of using small rodents as cancer models is their non-human metabolism. While their metabolic principles are comparable to those of humans, the metabolic rate of small rodents is often much higher [5].

**Figure 4:**
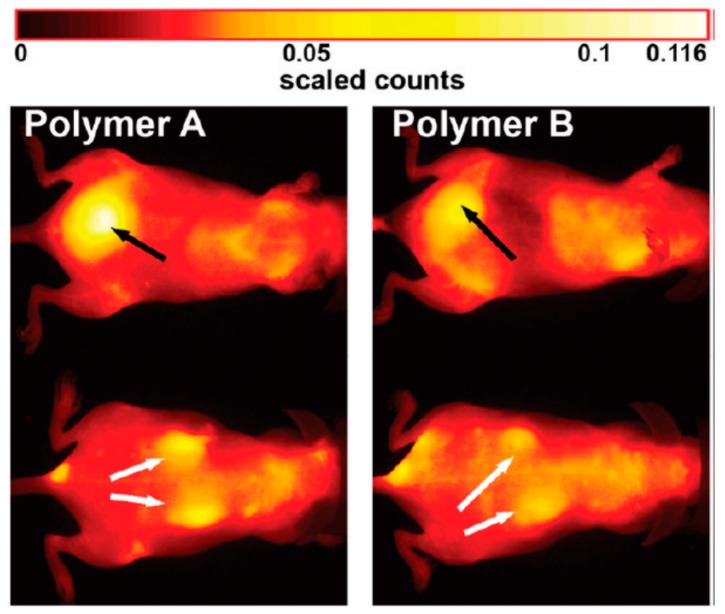
Distribution of HPMA-based copolymers in healthy SKH-1 mice 6 h after *i.v.* administration of 1 mg linear HPMA (30 kDa, polymer A) or star-like HPMA (200 kDa, polymer B) in dorsal and abdominal images. Arrows mark bladder (black) and kidneys (white). Reprinted with permission from [31]. Copyright [2012] American Chemical Society.

The analysis of in vivo FRI data is critical because the quantification of fluorescence intensities is difficult. To illustrate the issue, we will take a brief look at *i.v.* administration of the fluorescently labeled HPMA-based copolymers. Figure 4 shows the typical results of a DDS pharmacokinetic study performed in healthy, hairless SKH-1 mouse with a Maestro^TM^ in-vivo fluorescence imaging system (CRi, Inc.; now PerkinElmer, Inc., Waltham, MA, USA). Both tested polymer carriers were based on synthetic water-soluble polymers and labeled with the NIR dye Dyomics DY-782. The pseudo-colored images of mice are displayed at selected time point—6 h after administration. Regarding the influence of the molecular weight, the larger star-like copolymer B showed a stronger fluorescence signal compared with the seven-times smaller linear copolymer A. A significantly higher fluorescence intensity (yellow color) of polymer A in kidneys indicated faster renal clearance compared with polymer B [31].

**Figure 5:**
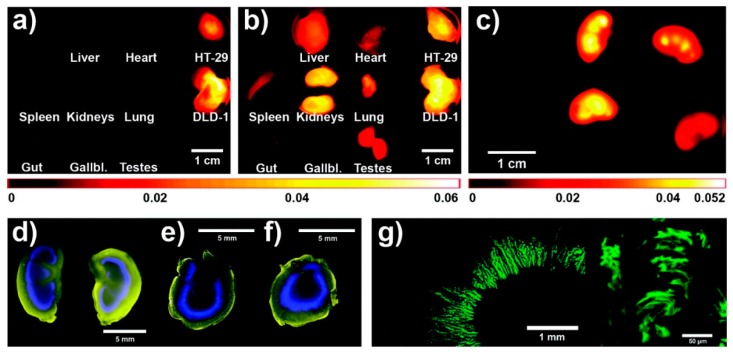
Ex vivo fluorescence images of organs and tumors: 2D-fluorescence reflectance imaging images of the model drug DY-676 (**a**) and HPMA copolymer (**b**) of mouse that was treated with star-like HPMA copolymer (polymer B); distribution of the model drug in kidneys 24 h after intravenous administration; left: placebo, middle: star-like HPMA, right: linear HPMA (**c**); pseudo-colored fluorescence images of kidney slices 24 h after *i.v.* injection—model drug: blue, HPMA polymer: yellow (**d**–**f**) (linear HPMA: d and e, star-like HPMA: **f**); Confocal microscopic images of the model drug distribution in the kidney 24 h after *i.v.* injection of 1.5 mg linear HPMA (polymer A) (**g**). Reprinted with permission from [31], Copyright [2012], American Chemical Society.

The tumor accumulation of both polymers was then similarly tested in athymic nude mice bearing two different human colorectal carcinoma xenografts (DLD-1 and HT-29). Since a passive tumor accumulation was apparent using FRI (data not shown), subsequent ex vivo analyses of necropsied organs/tumors provided information about the DDS biodistribution in the body (see Figure 5). It is clearly visible that both polymers were accumulated within both tumors and kidneys, confirming the enhanced tumor accumulation and elimination via renal filtration.

**Figure 6:**
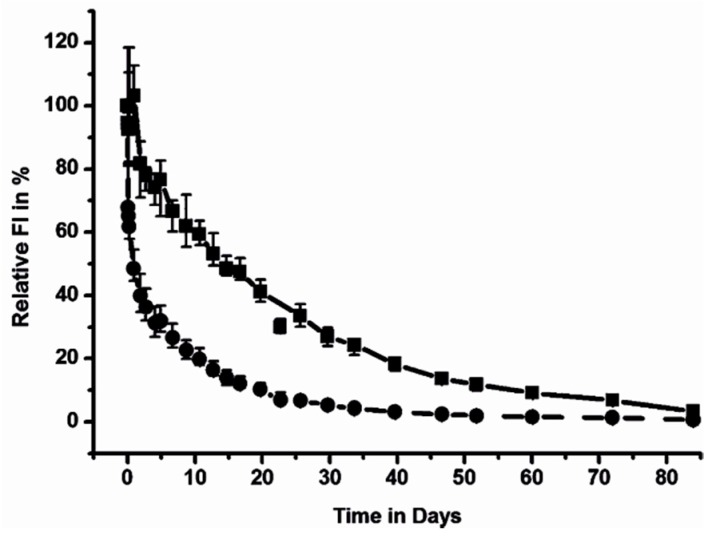
Decrease in the overall fluorescence intensity of HPMA-based copolymers after *i.v.* administration of 1 mg polymer into healthy SKH-1 mice (**●──**linear copolymer with *M*_w_ = 30,000 g/mol; **■──**star copolymer with *M*_w_ = 200,000 g/mol). Reprinted with permission from [31]., Copyright [2012], American Chemical Society. Recently, an effective relative method for the evaluation of tumor uptake of DDS was published. The authors defined a tumor accumulation value (TAV) [31] based on the calculation of unmixed grayscale images of the single spectral species based on the fluorescence intensity (I) and fluorescent area. Here, signals from the tumor (I_tumor_) and the remaining healthy areas of the mice (I_mouse_ − I_tumor_) were compared according the following equation: TAV = (proportion of I)/(proportion of area) = [I_tumor_/(I_mouse_ − I_tumor_)]/ [area_tumor_/(area_mouse_ − area_tumor_)]. The advantage of the TAV method consists of the elimination of intensity fluctuations and differences within different regions of the tumor. However, use of the TAV calculation in combination with NIR fluorescent dye accumulation in tumors is even underestimated because NIR light is more commonly scattered in the tissues than absorbed [4]. Thus, the more intensive I_tumor_ is also scattered throughout the whole mouse body, and thus the mouse body seems to be brighter than it really is. Therefore, a higher DDS accumulation in solid tumor than calculated can be expected [31]. An illustrative example of the TAV calculated for NIR-labeled hydroxyethyl starch accumulation in carcinoma xenografts is shown in Figure 7 [86].

All the results shown above were based solely on a comparison of fluorescent intensities without any relative or even absolute quantification. However, such information would be highly fundamental for comparisons of different DDS with varying doses in different animals at various time points. In the case of a relative quantification, data obtained from identical individuals and experimental settings are compared at fixed time points. Thus, it is possible to compare the data points of one group with those of the other (Figure 6). In detail, the relative total fluorescence intensities of whole mice over time revealed differences between the elimination of polymers from the mouse body. The high-molecular-weight star polymer B was detectable in the mouse for more than 10 weeks, which was significantly longer than the linear polymer. Similarly, the fluorescence intensities for selected organs or tumors could be compared.

Various DDS pharmacokinetics were studied using FRI, among others nanoparticles. Recently, an interesting review article described indocyanine green (ICG) application in the OI. ICG is a Food and Drug Administration (FDA)-approved NIR fluorescent dye, utilized in various biomedical applications such as drug delivery, imaging, and diagnosis, due to its attractive physicochemical properties, high sensitivity, and better imaging view field. Various polymer materials, poly(d,l-lactic-*co*-glycolic acid) (PLGA), poly(ethylene glycol) (PEG), and poly(ε-caprolactone) (PCL) were labeled or ICG was encapsulated to their nanoparticles to follow the biodistribution or selective accumulation by OI [87]. For example, Kolitz-Domb et al. [88] studied the use of ICG as a NIR label for proteinoid-poly(l-lactic acid) nanoparticles as a model of diagnostics. Their data confirmed that the particles were easily eliminated from the body within 24 h after *i.v.* administration. Furthermore, Benoît et al. presented the biodistribution data for lipid fluorescently labeled nano-capsules and compared their FRI results with MRI. They demonstrated that both techniques revealed the same findings [89].

**Figure 8:**
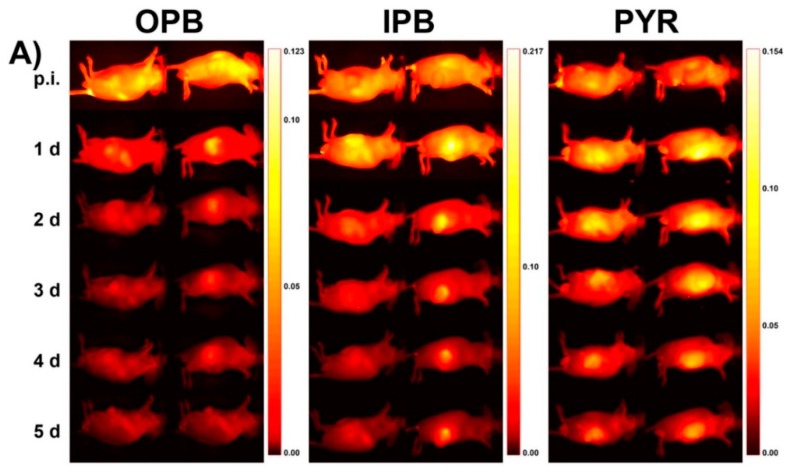

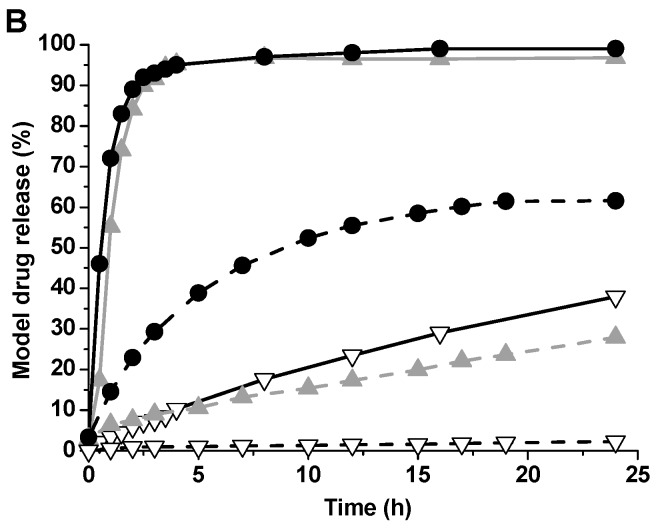
(**A**) Drug model (fluorescent dye DY-676) distribution in human colon carcinoma-bearing nude mice after injection of star-like HPMA-based copolymers containing different spacers—4-(2-oxopropyl)benzoyl (OPB), 4-isopropyl-4-oxobutanoyl (IPB), and 4-oxo-4-(2-pyridyl)butanoyl (PYR) spacers. (Reprinted with permission from [32], Copyright [2017], Elsevier). (**B**) Release of drug model from the copolymers incubated in phosphate buffered saline at pH 5.0 (**●──**OPB; ▲**──**IPB; ▽**──**PYR) and pH 7.4 (**●**- - - OPB; ▲- - - IPB; ▽- - - PYR) at 37 °C.

FRI enables not only the study of the pharmacokinetics of polymer carriers but also, simultaneously, the acquisition of an understanding of the fate of the carried drug in the body. Dual-labeled fluorescent polymer systems have recently been described in literature [31,32]. In detail, the linear or star-like HPMA-based polymer carriers were labeled via a stable covalent bond with the NIR dye Dyomics DY-782 and concurrently also with the far red fluorescent dye Dyomics DY-676 bound to the polymer carrier by a pH-sensitive hydrazone bond [31]. The far red dye acted as a model drug designed for pH-sensitive release and thus activation in tumor tissue/cells, while the NIR dye enabled monitoring of the polymer carriers. Since the excitation and emission wavelengths of the dyes were far away, more than 100 nm, the pharmacokinetics of both dyes could be observed simultaneously to compare the biodistribution of the carriers and the carried drug. Moreover, a subsequent work was focused on the impact of the drug release rate on tumor accumulation. Similar dual fluorescently labeled HPMA-based copolymers differing in the structure of the pH-sensitive spacer were compared using FRI [32]. The drug release rate was found to play a very important role in the overall drug circulation time and accumulation within the tumor mass. Due to the very rapid release of far red dye bound to the 4-(2-oxopropyl)benzoyl (OPB) spacer from the polymer carrier at blood pH (62% of the released dye within 24 h at pH 7.4, 37 °C), only a minor accumulation of the drug model was observed in tumor tissue (see the distribution of the dye in tumor-bearing mice in vivo and the dye release rates in vitro shown in Figure 8). Tumor accumulation of the dye bound to the 4-isopropyl-4-oxobutanoyl (IPB) also revealed that a slower dye release (28% within 24 h at pH 7.4) was not effective, although it was higher when compared with the OPB spacer. The highest accumulation of dye in tumor was observed for the dye bound to the 4-oxo-4-(2-pyridyl)butanoyl (PYR) spacer, which ensured only negligible dye release at blood pH (2% within 24 h at pH 7.4). This effect was independent of the carrier structure and molecular weight. In this case, FRI was able to confirm the crucial relationship between the structure of the DDS and the final biological outcome, thus facilitating the design of more potent polymer therapeutics.

Recently, dual fluorescently labeled HPMA-based copolymers with a polymer carrier labeled with NIR dye and bearing a model drug, fluorescent far red dye, bound to either the reductively degradable disulfide bond [90] or enzymatically degradable oligopeptide Gly-Phe-Leu-Gly spacer, [91] were examined in detail by FRI. In addition to the biodistribution of both, the polymer carrier and model of the drug determination confirmed that the fluorescent models of the drugs were specifically cleaved inside the tumors. In addition, FRI was able to validate that disulfide or enzymatically degradable oligopeptides are suitable biodegradable linkers between the drug and polymer carrier in DDS that can be exploited for the treatment of solid tumors. The comparison of FRI with PET was evaluated recently using ^89^Zr- or Dyomics676-labeled HPMA-copolymer conjugates differing in molecular weight with either low dispersity or high dispersity [64]. In vivo and ex vivo data obtained from PET were in very good agreement with observation by FRI. Both techniques showed that dispersity and molecular weight of the linear HPMA polymer carriers have a significant influence on the in vivo fate of the polymer conjugates.

The combination of therapy and simultaneous diagnostics, abbreviated as theranostics, has become very attractive in recent years. FRI could be a perfect tool for the observation of pharmacokinetics (see previous section) and for the visualization of the therapy and its effects on the organism.

**Figure 9:**
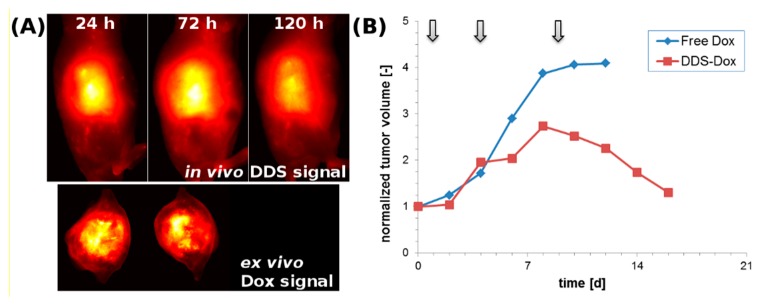
(**A**) Top, lateral mouse images of a subcutaneous growing xenograft tumor 24, 72, and 120 h after the first drug delivery system (DDS) administration. The in vivo tumor accumulation of the NIR fluorescently labeled DDS (containing Dox attached via a pH-sensitive hydrazone bond) is clearly visible (yellow to white area). Bottom, a tumor cross-section showing a central area with high Dox accumulation. (**B**) The plot displays data from mice bearing a Dox-resistant subcutaneous xenograft tumor treated with free Dox (blue curve) or with the DDS loaded with Dox (red curve). Therapy application is indicated by the grey arrows. It is clear that free Dox cannot initiate tumor regression, whereas DDS-delivered Dox can significantly reduce tumor volume after the third injection. This remission is induced by therapeutic intratumoral Dox levels caused by the enhanced permeability and retention effect-based DDS accumulation. Reprinted with permission from [5], Copyright [2016], Elsevier.) Potential of poly(ethylene glycol)-block-poly(d,l-lactic acid) (PEG-b-PLA) micelles carrying a carbocyanine dye (1,1′-dioctadecyl tetramethyl indotricarbocyanine iodide, DiR) for tumor-primed NIR optical imaging for intraoperative surgical guidance in oncology was evaluated in detail [92]. Use of labeled PEG-b-PLA micelles resulted after 48 h in a 2.1-fold higher NIR optical signal from excised solid tumors versus a negative control, presumably due to a reduction in tumor cell density and interstitial tumor pressure. Similarly, HPMA-based polymer systems labeled with a fluorescent dye Dy-633 or Cy-7 and decorated with targeting oligopeptides GE-7 or GE-11, specific targeting ligands binding to epidermal growth factor receptor (EGFR) highly expressed on surface of tumor cells, were described. The polymer probes targeted by the GE-11 oligopeptide were found in vivo as highly effective in tumor accumulation, as determined from OI. Indeed, the ex vivo cross-section of the tumors showed significant tumor border fluorescence proving the potential of the studied polymer probes for fluorescence-guided optical surgery of tumors [93].

The combination of a NIR dye and a drug to the DDS provides diagnostic and therapeutic applications in a single system. The tumor therapy using labeled HPMA-based polymer-drug conjugates containing Dox bound to a pH-sensitive spacer is a nice example of the application of FRI within theranostics [43]. Using NIR labeling, the biodistribution and tumor accumulation of the polymer conjugate can be visualized in vivo (Figure 9). Because Dox is a self-fluorescent drug, it can also be visualized by FRI. Nevertheless, due to its spectral properties (Ex/Em of 480/590 nm), only ex vivo imaging can be achieved (Figure 9). The therapeutic efficacy of the Dox-containing DDS in a Dox-resistant model was monitored by caliper measurement and tumor volume calculation (Figure 9B). Using the mentioned labeled DDS, the pharmacokinetics together with the treatment efficacy could be followed and thus the treatment schedule adjusted based on the DDS pharmacokinetics in the body and the tumor volume.

Indeed, the pH-sensitive poly(ethylene glycol)-*block*-(β-amino ester) (PEG-*b*-PAE) micelles have been utilized to deliver optical imaging agents as a pH-sensitive nanoflash for acidic tumoral imaging [94]. Tetramethylrhodamine (TRITC)-loaded PEG-*b*-PAE micelles exhibit self-quenched fluorescence at pH 7.4 due to the high local loading density. Quenching is eliminated and the fluorescence recovers at low pH due to pH-induced demicellization, the release of the cargo, and the local dilution of the dye. In vivo studies have shown that TRITC-loaded PEG-*b*-PAE micelles can successfully deliver TRITC to a tumor site and release it there, resulting in discernible fluorescence at the tumor site [95].

**Figure 10:**
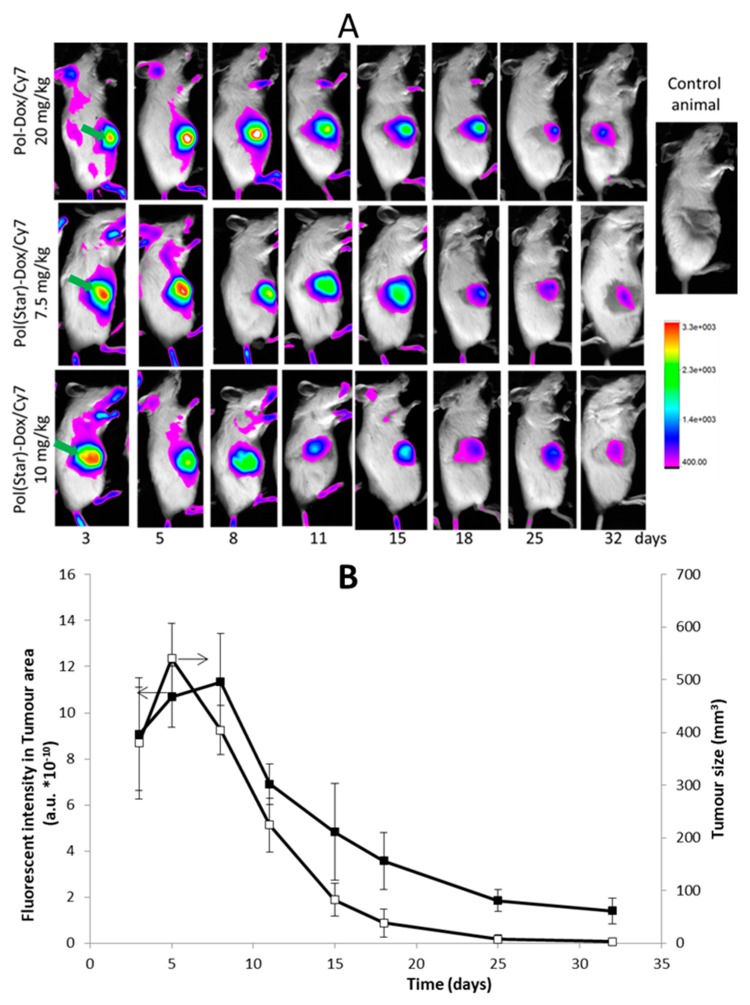
(**A**) In vivo optical imaging. Serial in vivo optical imaging of RAJI tumour-bearing mice injected with linear polymer Pol-Dox/Cy7 (dose 20 mg Dox eq./kg), star polymer Pol(Star)-Dox/Cy7 (dose 7.5 and 10 mg Dox eq./kg) at 3, 5, 8, 11, 18, 25, and 32 days intraperitoneally. Green arrows indicate the position of RAJI tumours; (**B**) comparison of the fluorescent intensity in the tumor area (left axis) and tumor size measured by the caliper (right axis) for treatment of RAJI-based xenografts with Cyanine7-labelled star-like HPMA-based polymer conjugates with Dox in the dose 7.5 mg/kg. The conjugate was administered *i.p.* at day 1, when all mice developed palpable *s.c.* tumors; (□──) tumor size; (■──) fluorescent intensity in tumor area. (Reprinted with permission from [97], Copyright [2017], Elsevier.

Recently, a theranostics potential of water-soluble NIR dye Cyanine7-labelled HPMA-based polymer-Dox conjugates was confirmed in treatment of various non-Hodgkin lymphomas [96]. The labelled polymer-Dox conjugates enabled concurrent evaluation of dynamic biodistribution and anti-lymphoma efficacy as highly therapeutically effective theranostics as is illustrated on Figure 10. Because of the longer circulation time, the star polymer drug carrier system appears to be the best candidate for the use as theranostics, showing the potential to treat and visualize the lymphoma tumors for at least one month after the injection. Due to limitation of FRI in deeply localized tumors, the theranostic potential can be utilized only in the case of subcutaneously localized tumors.

In addition to chemotherapy, photodynamic therapy (PDT) is a powerful tool in cancer treatment. Its principle consists of the production of cytotoxic substances (namely singlet oxygen species) after light induction of a photosensitizer located in the treated tissue. Porphyrin derivatives are often used with superiority as photosensitizers due to their spectral properties [97]. Because of their fluorescence in the far red spectrum, their biodistribution can be visualized by FRI. For example, the use of HPMA-based polymer micelles with zinc protoporphyrin enabled the simultaneous PDT as well as polymer biodistribution and tumor accumulation [98,99].

## 4. Fluorescence-Based Tomography and Future Prospects

Universally, FRI is accepted as highly efficient tool for the imaging of nano-scaled DDS pharmacokinetics in surface tissues, such as subcutaneously inoculated tumors. FRI has been used successfully for comparative analyses between the DDS intended for cell-specific drug targeting or EPR-mediated drug targeting to solid tumors. Unfortunately, a detailed quantitative analysis and accumulation and pharmacokinetics in deep tissues cannot be determined solely by FRI [28]. The precise determination of absolute amounts of accumulated DDS within tumors or in physiologically relevant healthy organs by FRI is rather complicated or impossible at this time. Recently, a large effort was applied to overcome this limitation by combining in vivo imaging with ex vivo tissue analysis with the aim of determining the exact dose percentage that could reach the targeted tissues.

**Figure 11:**
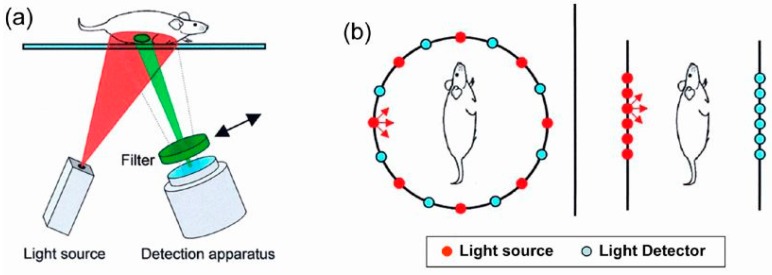
(**a**) Schematic of an epi-luminescence 2D-fluorescence reflectance imaging system that is commonly used for macroscopic fluorescence imaging applications but suffers from poor resolution and nonlinear signal attenuation in deep tissues. (**b**) Schematic of FMT systems using ring or planar geometry. Multiple source (red dots) and detector (blue dots) projections are made through the animal, and the fluorescence distribution is calculated by back-projecting physical models of light propagation through tissue. Reprinted from [100]

Recent improvements in optical technology have moved fluorescent imaging beyond the standard two-dimensional epifluorescence imaging into the domain of three-dimensional (3D) fluorescence molecular tomography (FMT) for improved quantification in deep tissue, see Figure 11. This procedure takes advantage of the trans-illumination of animals (i.e., the passing of light through the animals) rather than the standard surface illumination used for epifluorescence imaging. The use of short pulses of light in a raster scan design has been suggested as a suitable OI method for retrieving the signal depth to enable three-dimensional imaging [101]. Alternatively, the depth-dependent attenuation of different wavelengths has been suggested as another property to exploit measurements of the depth of fluorophores in the body [102]. This advance introduced by fluorescence tomography is accompanied by the need for extra care in performing proper imaging, e.g., animals must be hair-less, must be properly injected with imaging agents for optimal delivery to imaging sites and minimization of artifacts, and scans must be set up and acquired under optimal conditions and settings. When carried out properly, deep tissue FMT with appropriate NIR imaging agents allows the detection and quantification of important biological processes, such as cellular protease activity, vascular leak, and receptor upregulation, by accurately reconstructing the in vivo distribution of systemically injected NIR imaging agents.

FMT is based on the 3D reconstruction of the fluorophore distribution in tissues based on light measurements collected at the tissue borders. In recent years, a growing number of studies that were focused on advanced reconstruction methods and single-pixel detection were published [103,104,105,106]. This technique is similar to that of X-ray computed tomography (CT, μCT) and is also frequently combined with this method [24]. In FMT, the tissue is exposed to light at different points or projections, and the collected emitted fluorescence light is used together with a sophisticated mathematical calculation that details the transport of photons within the tissues. Lasers excite NIR fluorophores in laboratory animals at up to 120 spatial locations, and consequently planar detectors, i.e., CCD chip cameras, detect the excitation and emission images of diffuse light propagations in animals. Finally, advanced mathematical algorithms reconstruct the 3D image of the optical imaging agent concentration within the examined animal. Allocation of the FMT signal to body compartment is usually based on the combination with μCT, which determines the 3D image of the animal body. In summary, FMT may be capable of overcoming the shortcomings associated with planar FRI and can enable more quantitative and in-depth analyses of NIR-labeled DDS in non-superficial tissues.

Excluding small-animal imaging, basic studies toward human clinical imaging have also been published. The approval of some useful fluorescent probes for human use in the field of fluorescent clinical imaging is expected to facilitate significant developments. It is typically believed that FMT will likely play a significant role in endoscopic methods [107]. Although other imaging techniques, such as CT, MRI, and PET, play a major role in accurate preoperative diagnostics, generally these techniques cannot be applied intraoperatively. In contrast, OI is a highly promising technique that provides a valuable sensitivity and specificity for tumor margin detection. Additionally, available clinical applications have confirmed that optical molecular imaging is a powerful intraoperative tool for guiding surgeons performing precision procedures, thus enabling radical resection and improved survival rates using contrast agents and surgical navigation systems. FMT can play a significant role in intraoperative imaging-guided cancer surgery. In this particular case, the exogenous fluorescence contrast could play a significant role in diagnostics by identifying the molecular onset of diseases and visualizing small disease foci, metastases and the real borders of the disease, which would otherwise be barely or even impossible to detect [108]. A highly valuable and promising application of FMT should be optical mammography. The detection of breast cancer is already a primary focus of optical tomography [109,110,111] because the human breast tissue is rather transparent to NIR light, thus allowing a high detection sensitivity and accuracy by optical imaging [112,113]. Finally, FMT can offer a valuable alternative to X-ray mammography to improve the detection specificity and increase the safety of the patient due to the absence of ionizing radiation. In this case, the FMT should be an optimal method to monitor drug efficacy and long-term treatment.

However, even FMT has some main limitation. The major disadvantage is based on its inability to accurately assign the reconstructed fluorescent signal to a given organ of interest [114,115]. This drawback can be overcome by combining FMT and μCT, thus combining the 3D fluorescence signal with the X-ray signal from the internal structure of a non-uniformly composed and opaque object (i.e., a non-transparent object of varying density and composition) in the human body, for molecular imaging purposes [116,117,118,119,120,121]. These studies verified and indicated the huge potential afforded by the combination of the very precise 3D organ and tissue determination of μCT, and the high fluorophore sensitivity of FMT within animal organs. The imaging performance and accuracy were considerably improved and accurate for the hybrid method compared with FMT alone.

**Figure 12:**
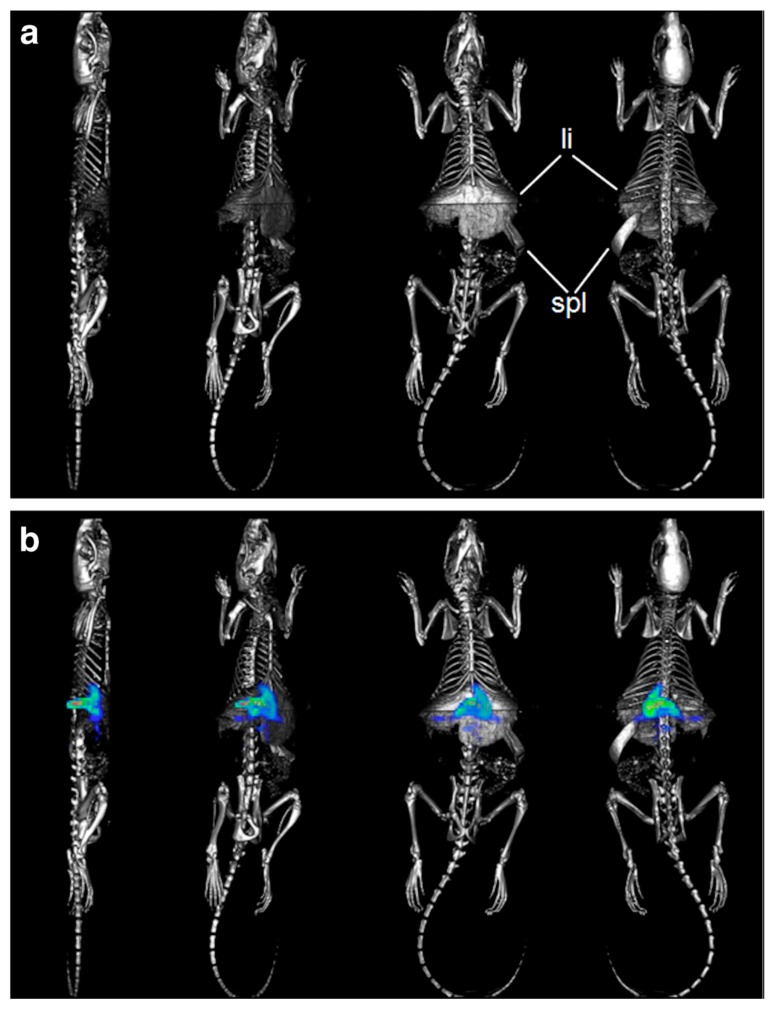
Accumulation of liver-targeted imaging agents 6 h after simultaneous injection with ExitronNano 1200 BSA_VT750. The animals (160 g Wistar rat; *n* = 1) were imaged in both modalities, and the scans were co-registered based on the fiducial marks. The 3D rendered volumes are shown in four different views rotated around the anteroposterior axis (second-last view: ventral, last view: dorsal). (**a**) Micro-CT, the barium sulfate nanoparticles accumulate in the liver (li) and the spleen (spl), resulting in enhanced X-ray contrast; (**b**) Micro-CT merged with FMT. Reprinted with permission from [118], Copyright [2014], Springer.

FMT is commonly applied for quantitative 3D imaging in mice. However, rats are also a highly relevant preclinical model, and thus FMT performance in rats was assessed based on the combination of FMT/μCT reconstructed data sets obtained from models that are relevant for tumor imaging, bone remodeling, and biodistribution analysis of nanoparticles [118]. Additionally, using the bone-targeting imaging agent Osteosense 750, regions of neo-bone formation were identified successfully by FMT. Finally, as a proof of the FMT/μCT concept in rats, nanoparticle DDS pharmacokinetics of the VT750–albumin conjugate were determined based on the accumulation/clearance in/from the liver at 11 different time points over 2 weeks (Figure 12). In conclusion, Vonwil and coworkers validated FMT imaging in 160 g rats, and sequential FMT/μCT imaging can be considered a useful tool for preclinical research in rats.

**Figure 13:**
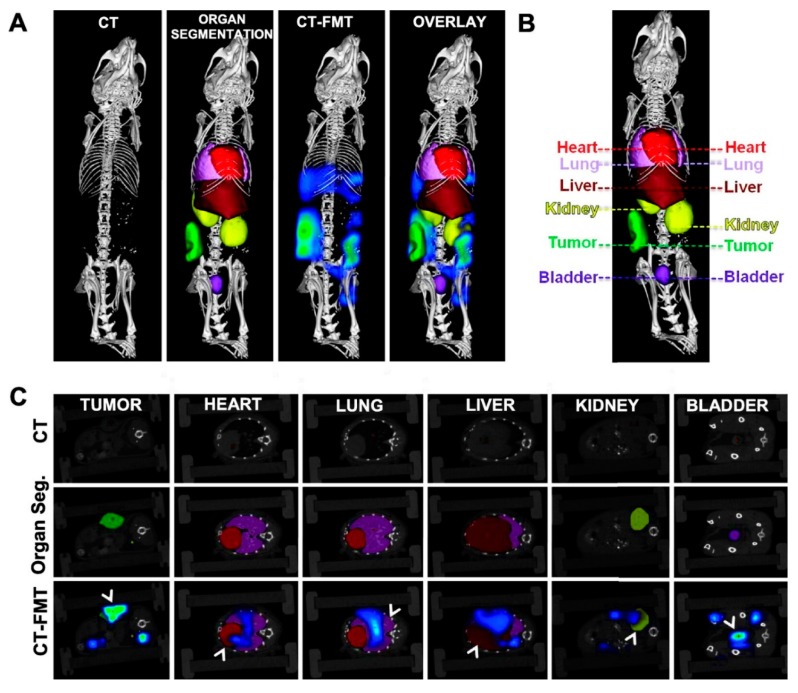
CT-based organ segmentation and hybrid CT-FMT imaging. (**A**) High-resolution μCT scans of CT26 colon carcinoma-bearing nude mice, depicting highly electron-dense anatomical structures (i.e., bones), presegmented organs (cf. panel C), FMT-based biodistribution data overlaid on highly electron-dense anatomical structures, and FMT-based biodistribution data overlaid on presegmented organs. (**B**,**C**) Two-dimensional planes representing individual organs (**B**) and pHPMA-Dy750 accumulation in cross-sections of these organs (**C**), analyzed by fusing μCT and FMT data sets. Reprinted with permission from [124], Copyright [2013], American Chemical Society.

Recently, the hybrid FMT-μCT imaging technique was used to determine the pharmacokinetics of nano-DDS formulations in tissues other than superficial or subcutaneous tumors [122]. The biodistribution and tumor accumulation of NIR dye-containing HPMA polymer carriers were observed and quantified using 3D FMT. The fluorescence-based data sets were then combined with 3D anatomical μCT data with several physiologically relevant pre-segmented organs (see Figure 13). Water-soluble NIR dye-labeled DDS were used to validate accumulation of DDS at the tumor and potentially in healthy organs and compare the results with those reported in the literature. Finally, these results confirmed that the combination of anatomical μCT with molecular FMT provided a powerful tool for the non-invasive biodistribution assessment of nanomedicines.

Similarly to the examples described above, water-soluble HPMA polymer actively targeted by oligopeptide containing an RGD or NGR sequence to angiogenesis-related surface receptors was evaluated and compared with a non-targeted polymer carrier using the hybrid FMT-μCT imaging method [123]. Polymeric nano-carriers with a size of 10 nm were synthesized and labeled with Dyomics-676 (targeted DDS) or with Dyomics-750 (non-targeted DDS) and co-injected into mice bearing both rapidly growing and highly leaky CT26 tumors and slowly growing and poorly leaky BxPC3 tumors. Using hybrid FMT-μCT, the authors concluded that vascular targeting was effective and resulted in rapid and efficient early binding to tumor blood vessels. Indeed, classical passive targeting based on the EPR effect was significantly more pronounced, enabling more efficient drug retention within the solid tumors. Although the results may vary for other types of tumors with different vascularization, the tissue structure and origin or other DDS based on micelles or liposomes, these insights indicate that the potential of active targeting should not be overestimated. The experiment also confirmed the unique potential of the hybrid FMT-μCT imaging method for solving such highly complex and significant issues associated with controlled drug delivery. Similarly, FMT was used to determine the biodistribution and targeting capacity of the anti-5T4 monoclonal antibody (mAb)-drug conjugates [124]. The results demonstrated that conjugation of the fluorescent dye VT680 to 5T4-mAb or 5T4-mAb-drug-conjugates did not change the behavior of the native biologic, and FMT imaging could be a useful tool to understand the biodistribution and tumor targeting kinetics of antibodies, mAb-drug-conjugates, and other biologics. Interestingly, Ma and coworkers described ανβ3 integrin targeted NIR fluorescence probe utilization with hybrid FMT/CT for monitoring tumor progression as well as the early therapy response in a syngeneic murine non-small cell lung cancer (NSCLC) model [121]. While μCT revealed only a moderate deceleration of tumor growth during the therapy regimen with cisplatin and bevacizumab, the ανβ3-dependent signal decreased significantly in comparison to the non-treated mice as soon as one week after treatment. Thus, the FMT/CT technique in combination with targeted imaging might become a promising tool for assessments of an early therapeutic response. Recently, HPMA polymer conjugates labelled by fluorophores ATTO 488-NH_2_ and Dy750-NH_2_ were employed to show that normalizing the tumour vasculature improves the accumulation of polymer carriers in tumours, and promotes their penetration out of tumour blood vessels deep into the interstitium [125].

Together with the water-soluble polymer DDS, the pharmacokinetic profiles of classical nanoparticles were determined using a hybrid FMT-CT imaging method [126]. In this case, fluorescently labeled self-assembled nucleic acid nanoparticles for targeted siRNA delivery were administered to mice. The nanoparticles were confirmed by hybrid imaging to circulate for a longer time in the blood stream than the parent siRNA and exhibited more profound accumulation in tumor xenograft mouse models, leading to enhanced gene silencing. Thus, FMT is also able to follow the distribution of gene delivery vectors.

In addition to water-soluble and self-assembly systems for siRNA delivery, the therapeutic potential of lipid nanoparticles tailored for siRNA delivery has been studied in detail using the hybrid FMT-CT method. Quantitative in vivo whole body imaging of the nanoparticles was determined using the FMT-CT system with fluorescently labeled siRNA [127]. As in the previous cases, the fluorescent signal equivalent to the siRNA concentration was clearly attributed to anatomical structures using CT data. Interestingly, the majority of the nanoparticles were accumulated in the spleen. In addition, FMT-CT imaging provided detailed information about the excretion pathway of lipid nanoparticles used as the siRNA carrier. The distribution determined by FMT-CT imaging was highly consistent with the plasma and tissue siRNA concentration derived from a PCR-based method of siRNA quantification. To assess the value of FMT-µCT scans of several representative fluorescent probes were acquired and the data were analyzed using organ segmentation [128]. FMT-µCT could differentiate between renal and hepatobiliary elimination and detect retention sites such as liver, kidney, spleen, bone, and lungs.

In addition to CT, other advanced “anatomical” imaging techniques have been combined with FMT, e.g., magnetic resonance [129,130,131], PET [132], and ultrasound [133,134,135]. The development of novel contrast agents (typically Gd-based) with enhanced relaxation times has strengthened magnetic resonance imaging for the characterization of functional tumor parameters such as pH, vascularization, and metabolism [136,137]. Indeed, the sensitivity of the contrast agents and the acquisition times remain rather limited. Nevertheless, tumor-targeted liposomes [138] and magnetic nanoparticles [139] with NIR-fluorescent and magnetic resonance probes containing siRNA have been studied in detail.

The application of fluorescent imaging agents that detect and quantify a variety of biological activities is already expanding the horizons of pre-clinical research and drug development. In summary, hybrid techniques that combine the highly efficient and practicable FMT tomography with other 3D tomography methods seem to be powerful quantitative tools for the 3D imaging of novel DDS in preclinical research and, hopefully in the future, also in clinical applications.

## 5. Conclusions

In the last decade, FI has grown into an integral and highly powerful part of drug delivery research, partly due to the potential of the method to facilitate collection of highly accurate pharmacokinetic data at reasonable costs and with easy-to-use instruments. Worldwide, FI has become a highly evolving method, as indicated by the enormously growing number of publications, applications and technological opportunities. This chapter has described current state-of-the-art advantages, drawbacks, and prospective technologies that can be used to perform in vitro, ex vivo and, above all, whole-body in vivo FI, even in 3D mode. Potential uses and limitations of FI, together with the advantages and disadvantages of different imaging techniques and the relevance of the data to used techniques are detailed here. A brief review of the literature presenting biological data obtained from this multi-faced FI-containing approach was introduced to document the real impact of FI on anti-cancer research. To date, because of the easy setup and low cost of the instrument, the vast majority of in vivo small animal imaging experiments are based on epi-illumination planar imaging. The future expansion of FI methods is highly dependent on the design of novel imaging systems based on the combination of state-of-art optical technology with highly precise structural modalities such as MRI or CT.

## Figures and Tables

**Figure 7:**
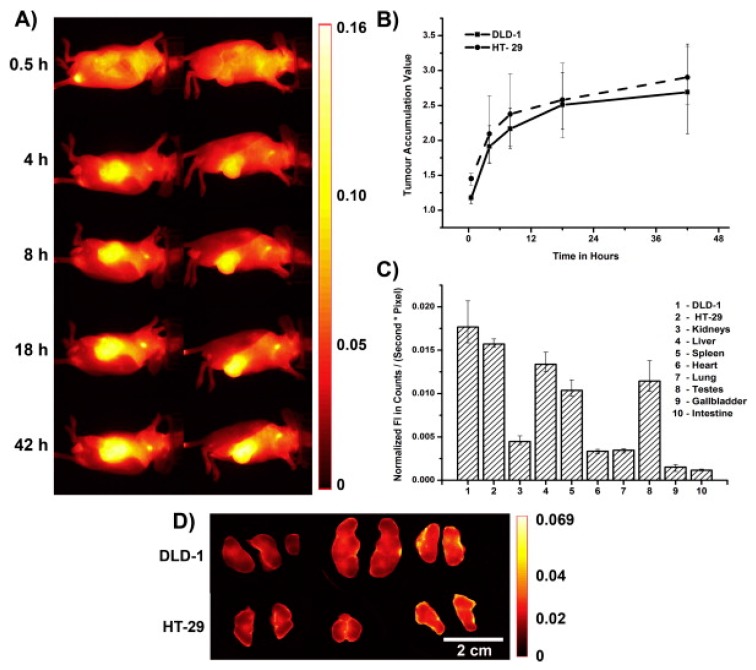
(**A**) In vivo images of colon carcinoma xenograft-bearing mouse after injection of 1.5 mg hydroxyethyl starch (HES) 450 kDa labeled with near infrared dye IR800CW. Left: HT-29, right DLD-1. (**B**) A comparable tumor accumulation value was calculated from the images for both tumors. (**C**) The fluorescence intensity measured from organs that were extracted 2 days after injection of HES 450. (**D**) Ex vivo images of autopsied xenograft colon carcinomas from 3 mice 2 days after injection of HES 450. Reprinted with permission from [87], Copyright [2013], Elsevier. Nevertheless, even the TAV calculation does not provide an absolute quantification. Values for the absolute intensities of fluorescent light have too many factors to be taken into account and may require additional studies. Such factors, encompassing the properties of particular DDS, the unique behavior of individual animals, and technique-independent parameters that influence the measured fluorescence intensity, among others, have been detailed in [5]. The detection of in vivo fluorescent signals for DDS in animal models for cancer therapy is quite easy using readily available instruments for fluorescence imaging if some basic conditions are considered. Regarding quantification, relative comparisons are possible. However, great care must be taken to achieve reliable absolute quantification. Some examples of DDS used in FRI are presented below.

## References

[1] Ulbrich K., Holá K., Šubr V., Bakandritsos A., Tuček J., Zbořil R. (2016). Targeted Drug Delivery with Polymers and Magnetic Nanoparticles: Covalent and Noncovalent Approaches, Release Control, and Clinical Studies. Chem. Rev..

[2] Boas D.A., Brooks D.H., Miller E.L., DiMarzio C.A., Kilmer M., Gaudette R.J., Zhang Q. (2001). Imaging the body with diffuse optical tomography. IEEE Signal. Proc. Mag..

[3] Gibson A.P., Hebden J.C., Arridge S.R. (2005). Recent advances in diffuse optical imaging. Phys. Med. Biol..

[4] Leblond F., Davis S.C., Valdes P.A., Pogue B.W. (2010). Pre-clinical whole-body fluorescence imaging: Review of instruments, methods and applications. J. Photochem. Photobiol. B.

[5] Etrych T., Lucas H., Janoušková O., Chytil P., Mueller T., Mäder K. (2016). Fluorescence optical imaging in anticancer drug delivery. J. Control. Release.

[6] Patra J.K., Das G., Fraceto L.F., Campos E.V.R., Rodriguez-Torres M.d.P., Acosta-Torres L.S., Diaz-Torres L.A., Grillo R., Swamy M.K., Sharma S. (2018). Nano based drug delivery systems: Recent developments and future prospects. J. Nanobiotechnol..

[7] Ganta S., Devalapally H., Shahiwala A., Amiji M. (2008). A review of stimuli-responsive nanocarriers for drug and gene delivery. J. Control. Release.

[8] Dozono H., Yanazume S., Nakamura H., Etrych T., Chytil P., Ulbrich K., Fang J., Arimura T., Douchi T., Kobayashi H. (2016). HPMA Copolymer-Conjugated Pirarubicin in Multimodal Treatment of a Patient with Stage IV Prostate Cancer and Extensive Lung and Bone Metastases. Target. Oncol..

[9] Duncan R., Gaspar R. (2011). Nanomedicine(s) under the Microscope. Mol. Pharm..

[10] Jain R.K., Stylianopoulos T. (2010). Delivering nanomedicine to solid tumors. Nat. Rev. Clin. Oncol..

[11] Maeda H., Wu J., Sawa T., Matsumura Y., Hori K. (2000). Tumor vascular permeability and the EPR effect in macromolecular therapeutics: A review. J. Control. Release.

[12] Matsumura Y., Maeda H. (1986). A New Concept for Macromolecular Therapeutics in Cancer-Chemotherapy - Mechanism of Tumoritropic Accumulation of Proteins and the Antitumor Agent Smancs. Cancer Res..

[13] Hoffman A.S. (2008). The origins and evolution of “controlled” drug delivery systems. J. Control. Release.

[14] Mulder W.J.M., Strijkers G.J., Van Tilborg G.A.F., Cormode D.P., Fayad Z.A., Nicolay K. (2009). Nanoparticulate Assemblies of Amphiphiles and Diagnostically Active Materials for Multimodality Imaging. Acc. Chem. Res..

[15] Ulbrich K., Šubr V. (2010). Structural and chemical aspects of HPMA copolymers as drug carriers. Adv. Drug Deliv. Rev..

[16] Venditto V.J., Szoka F.C. (2013). Cancer nanomedicines: So many papers and so few drugs!. Adv. Drug Deliv. Rev..

[17] Kunjachan S., Jayapaul J., Mertens M.E., Storm G., Kiessling F., Lammers T. (2012). Theranostic Systems and Strategies for Monitoring Nanomedicine-Mediated Drug Targeting. Curr. Pharm. Biotechnol..

[18] Lammers T., Aime S., Hennink W.E., Storm G., Kiessling F. (2011). Theranostic Nanomedicine. Acc. Chem. Res..

[19] Phillips M.A., Gran M.L., Peppas N.A. (2010). Targeted nanodelivery of drugs and diagnostics. Nano. Today.

[20] Allmeroth M., Moderegger D., Biesalski B., Koynov K., Rosch F., Thews O., Zentel R. (2011). Modifying the Body Distribution of HPMA-Based Copolymers by Molecular Weight and Aggregate Formation. Biomacromolecules.

[21] Lammers T., Kuhnlein R., Kissel M., Šubr V., Etrych T., Pola R., Pechar M., Ulbrich K., Storm G., Huber P. (2005). Effect of physicochemical modification on the biodistribution and tumor accumulation of HPMA copolymers. J. Control. Release.

[22] Lu Z.R. (2010). Molecular imaging of HPMA copolymers: Visualizing drug delivery in cell, mouse and man. Adv. Drug Deliv. Rev..

[23] Licha K., Olbrich C. (2005). Optical imaging in drug discovery and diagnostic applications. Adv. Drug Deliv. Rev..

[24] Ntziachristos V. (2006). Fluorescence molecular imaging. Annu. Rev. Biomed. Eng..

[25] Ke S., Wen X.X., Gurfinkel M., Charnsangavej C., Wallace S., Sevick-Muraca E.M., Li C. (2003). Near-infrared optical imaging of epidermal growth factor receptor in breast cancer xenografts. Cancer Res..

[26] Wunder A., Tung C.H., Muller-Ladner U., Weissleder R., Mahmood U. (2004). In vivo imaging of protease activity in arthritis—A novel approach for monitoring treatment response. Arthritis Rheum..

[27] Zaheer A., Lenkinski R.E., Mahmood A., Jones A.G., Cantley L.C., Frangioni J.V. (2001). In vivo near-infrared fluorescence imaging of osteoblastic activity. Nat. Biotechnol..

[28] Ntziachristos V., Ripoll J., Wang L.H.V., Weissleder R. (2005). Looking and listening to light: The evolution of whole-body photonic imaging. Nat. Biotechnol..

[29] Hebden J.C., Arridge S.R., Delpy D.T. (1997). Optical imaging in medicine.1. Experimental techniques. Phys. Med. Biol..

[30] Mahmood U., Weissleder R. (2003). Near-infrared optical imaging of proteases in cancer. Mol. Cancer Ther..

[31] Hoffmann S., Vystrčilová L., Ulbrich K., Etrych T., Caysa H., Mueller T., Mäder K. (2012). Dual Fluorescent HPMA Copolymers for Passive Tumor Targeting with pH-Sensitive Drug Release: Synthesis and Characterization of Distribution and Tumor Accumulation in Mice by Noninvasive Multispectral Optical Imaging. Biomacromolecules.

[32] Chytil P., Hoffmann S., Schindler L., Kostka L., Ulbrich K., Caysa H., Mueller T., Mäder K., Etrych T. (2013). Dual fluorescent HPMA copolymers for passive tumor targeting with pH- sensitive drug release II: Impact of release rate on biodistribution. J. Control. Release.

[33] Pu Y., Tang R., Xue J., Wang W.B., Xu B., Achilefu S. (2014). Synthesis of dye conjugates to visualize the cancer cells using fluorescence microscopy. Appl. Opt..

[34] Rodríguez-Rodríguez H., Acebrón M., Iborra F.J., Arias-Gonzalez J.R., Juárez B.H. (2019). Photoluminescence Activation of Organic Dyes via Optically Trapped Quantum Dots. ACS Nano.

[35] Xiong J., Cao X., Yang S., Mo Z., Wang W., Zeng W. (2018). Fluorescent Probes for Detection of Protein: From Bench to Bed. Protein Pept. Lett..

[36] Kumar S., Richards-Kortum R. (2006). Optical molecular imaging agents for cancer diagnostics and therapeutics. Nanomedicine-Uk.

[37] Freidus L.G., Pradeep P., Kumar P., Choonara Y.E., Pillay V. (2018). Alternative fluorophores designed for advanced molecular imaging. Drug Discov. Today.

[38] Gao X.H., Nie S.M. (2003). Molecular profiling of single cells and tissue specimens with quantum dots. Trends Biotechnol..

[39] Xue J.P., Shan L.L., Chen H.Y., Li Y., Zhu H.Y., Deng D.W., Qian Z.Y., Achilefu S., Gu Y.Q. (2013). Visual detection of STAT5B gene expression in living cell using the hairpin DNA modified gold nanoparticle beacon. Biosens. Bioelectron..

[40] Hoffman R.M. (2015). Application of GFP imaging in cancer. Lab. Investig..

[41] McCann T., Kosaka N., Choyke P., Kobayashi H., Hoffman R.M. (2012). The Use of Fluorescent Proteins for Developing Cancer-Specific Target Imaging Probes. In Vivo Cellular Imaging Using Fluorescent Proteins.

[42] Karasev M.M., Stepanenko O.V., Rumyantsev K.A., Turoverov K.K., Verkhusha V.V. (2019). Near-Infrared Fluorescent Proteins and Their Applications. Biochem-Moscow.

[43] Heinrich A.K., Lucas H., Schindler L., Chytil P., Etrych T., Mäder K., Mueller T. (2016). Improved Tumor-Specific Drug Accumulation by Polymer Therapeutics with pH-Sensitive Drug Release Overcomes Chemotherapy Resistance. Mol. Cancer Ther..

[44] Dolloff N.G., Ma X.H., Dicker D.T., Humphreys R.C., Li L.Z., El-Deiry W.S. (2011). Spectral imaging-based methods for quantifying autophagy and apoptosis. Cancer Biol. Ther..

[45] Galateanu B., Hudita A., Negrei C., Ion R.M., Costache M., Stan M., Nikitovic D., Hayes A.W., Spandidos D.A., Tsatsakis A.M. (2016). Impact of multicellular tumor spheroids as an in vivo-like tumor model on anticancer drug response. Int. J. Oncol..

[46] Ballou B., Fisher G.W., Hakala T.R., Farkas D.L. (1997). Tumor detection and visualization using cyanine fluorochrome-labeled antibodies. Biotechnol. Progr..

[47] Lidický O., Janoušková O., Strohalm J., Alam M., Klener P., Etrych T. (2015). Anti-Lymphoma Efficacy Comparison of Anti-Cd20 Monoclonal Antibody-Targeted and Non-Targeted Star-Shaped Polymer-Prodrug Conjugates. Molecules.

[48] Folli S., Westermann P., Braichotte D., Pelegrin A., Wagnieres G., van den Bergh H., Mach J.P. (1994). Antibody-indocyanin conjugates for immunophotodetection of human squamous cell carcinoma in nude mice. Cancer Res..

[49] Pechar M., Pola R., Janoušková O., Sieglová I., Král V., Fábry M., Tomalová B., Kovář M. (2018). Polymer Cancerostatics Targeted with an Antibody Fragment Bound via a Coiled Coil Motif: In Vivo Therapeutic Efficacy against Murine BCL1 Leukemia. Macromol. Biosci..

[50] Pola R., Studenovsky M., Pechar M., Ulbrich K., Hovorka O., Vetvicka D., Rihova B. (2009). HPMA-copolymer conjugates targeted to tumor endothelium using synthetic oligopeptides. J. Drug Target..

[51] Studenovsky M., Pola R., Pechar M., Etrych T., Ulbrich K., Kovar L., Kabesova M., Rihova B. (2012). Polymer carriers for anticancer drugs targeted to EGF receptor. Macromol. Biosci..

[52] Song Y., Zhu Z., An Y., Zhang W., Zhang H., Liu D., Yu C., Duan W., Yang C.J. (2013). Selection of DNA aptamers against epithelial cell adhesion molecule for cancer cell imaging and circulating tumor cell capture. Anal. Chem..

[53] Tung C.H. (2004). Fluorescent peptide probes for in vivo diagnostic imaging. Biopolymers.

[54] Weissleder R. (1999). Molecular imaging: Exploring the next frontier. Radiology.

[55] Shi H., Lei Y., Ge J., He X., Cui W., Ye X., Liu J., Wang K. (2019). A Simple, pH-Activatable Fluorescent Aptamer Probe with Ultralow Background for Bispecific Tumor Imaging. Anal. Chem..

[56] Muller-Taubenberger A., Anderson K.I. (2007). Recent advances using green and red fluorescent protein variants. Appl. Microbiol. Biotechnol..

[57] Hoffman R.M. (2005). The multiple uses of fluorescent proteins to visualize cancer in vivo. Nat. Rev. Cancer.

[58] Chudakov D.M., Matz M.V., Lukyanov S., Lukyanov K.A. (2010). Fluorescent Proteins and Their Applications in Imaging Living Cells and Tissues. Physiol. Rev..

[59] Choy G., Choyke P., Libutti S.K. (2003). Current Advances in Molecular Imaging: Noninvasive in Vivo Bioluminescent and Fluorescent Optical Imaging in Cancer Research. Mol. Imaging.

[60] Barua S., Yoo J.W., Kolhar P., Wakankar A., Gokarn Y.R., Mitragotri S. (2013). Particle shape enhances specificity of antibody-displaying nanoparticles. PNAS.

[61] Gratton S.E., Ropp P.A., Pohlhaus P.D., Luft J.C., Madden V.J., Napier M.E., DeSimone J.M. (2008). The effect of particle design on cellular internalization pathways. PNAS.

[62] Huang X., Teng X., Chen D., Tang F., He J. (2010). The effect of the shape of mesoporous silica nanoparticles on cellular uptake and cell function. Biomaterials.

[63] Shi J., Choi J.L., Chou B., Johnson R.N., Schellinger J.G., Pun S.H. (2013). Effect of polyplex morphology on cellular uptake, intracellular trafficking, and transgene expression. ACS nano.

[64] Koziolová E., Goel S., Chytil P., Janoušková O., Barnhart T.E., Cai W., Etrych T. (2017). A tumor-targeted polymer theranostics platform for positron emission tomography and fluorescence imaging. Nanoscale.

[65] Pola R., Laga R., Ulbrich K., Sieglová I., Král V., Fábry M., Kabešová M., Kovář M., Pechar M. (2013). Polymer Therapeutics with a Coiled Coil Motif Targeted against Murine BCL1 Leukemia. Biomacromolecules.

[66] Pola R., Král V., Filippov S.K., Kaberov L., Etrych T., Sieglová I., Sedláček J., Fábry M., Pechar M. (2019). Polymer Cancerostatics Targeted by Recombinant Antibody Fragments to GD2-Positive Tumor Cells. Biomacromolecules.

[67] Jiang S., Gnanasammandhan M.K., Zhang Y. (2010). Optical imaging-guided cancer therapy with fluorescent nanoparticles. J. R. Soc. Interface.

[68] Koziolová E., Machová D., Pola R., Janoušková O., Chytil P., Laga R., Filippov S.K., Šubr V., Etrych T., Pechar M. (2016). Micelle-forming HPMA copolymer conjugates of ritonavir bound via a pH-sensitive spacer with improved cellular uptake designed for enhanced tumor accumulation. J. Mater. Chem. B.

[69] Hovorka O., Etrych T., Šubr V., Strohalm J., Ulbrich K., Říhová B. (2006). HPMA based macromolecular therapeutics: Internalization, intracellular pathway and cell death depend on the character of covalent bond between the drug and the peptidic spacer and also on spacer composition. J. Drug Target..

[70] Machová D., Koziolová E., Chytil P., Venclíková K., Etrych T., Janoušková O. (2018). Nanotherapeutics with suitable properties for advanced anticancer therapy based on HPMA copolymer-bound ritonavir via pH-sensitive spacers. Eur. J. Pharm. Biopharm..

[71] Chen Y., Walsh R.J., Arriaga E.A. (2005). Selective determination of the doxorubicin content of individual acidic organelles in impure subcellular fractions. Anal. Chem..

[72] Shen F., Chu S., Bence A.K., Bailey B., Xue X., Erickson P.A., Montrose M.H., Beck W.T., Erickson L.C. (2008). Quantitation of doxorubicin uptake, efflux, and modulation of multidrug resistance (MDR) in MDR human cancer cells. J. Pharmacol. Exp. Ther..

[73] Priem B., Tian C., Tang J., Zhao Y., Mulder W.J.M. (2015). Fluorescent nanoparticles for the accurate detection of drug delivery. Expert Opin. Drug Deliv..

[74] Nori A., Kopecek J. (2005). Intracellular targeting of polymer-bound drugs for cancer chemotherapy. Adv. Drug Deliv. Rev..

[75] Chytil P., Koziolová E., Janoušková O., Kostka L., Ulbrich K., Etrych T. (2015). Synthesis and Properties of Star HPMA Copolymer Nanocarriers Synthesised by RAFT Polymerisation Designed for Selective Anticancer Drug Delivery and Imaging. Macromol. Biosci..

[76] Laga R., Janoušková O., Ulbrich K., Pola R., Blažková J., Filippov S.K., Etrych T., Pechar M. (2015). Thermoresponsive Polymer Micelles as Potential Nanosized Cancerostatics. Biomacromolecules.

[77] Braunová A., Kostka L., Sivák L., Cuchalová L., Hvězdová Z., Laga R., Filippov S., Černoch P., Pechar M., Janoušková O. (2017). Tumor-targeted micelle-forming block copolymers for overcoming of multidrug resistance. J. Control. Release.

[78] Zhang R., Yang J., Radford D.C., Fang Y., Kopeček J. (2017). FRET Imaging of Enzyme-Responsive HPMA Copolymer Conjugate. Macromol. Biosci..

[79] Yang J.Y., Zhang R., Radford D.C., Kopecek J. (2015). FRET-trackable biodegradable HPMA copolymer-epirubicin conjugates for ovarian carcinoma therapy. J. Control. Release.

[80] Fan W., Shi W., Zhang W., Jia Y., Zhou Z., Brusnahan S.K., Garrison J.C. (2016). Cathepsin S-cleavable, multi-block HPMA copolymers for improved SPECT/CT imaging of pancreatic cancer. Biomaterials.

[81] Bhuckory S., Kays J.C., Dennis A.M. (2019). In Vivo Biosensing Using Resonance Energy Transfer. Biosensors.

[82] Basuki J.S., Duong H.T.T., Macmillan A., Erlich R.B., Esser L., Akerfeldt M.C., Whan R.M., Kavallaris M., Boyer C., Davis T.P. (2013). Using Fluorescence Lifetime Imaging Microscopy to Monitor Theranostic Nanoparticle Uptake and Intracellular Doxorubicin Release. ACS Nano.

[83] Dai X.W., Yue Z.L., Eccleston M.E., Swartling J., Slater N.K.H., Kaminski C.F. (2008). Fluorescence intensity and lifetime imaging of free and micellar-encapsulated doxorubicin in living cells. Nanomed-Nanotechnol..

[84] Mansfield J.R., Gossage K.W., Hoyt C.C., Levenson R.M. (2005). Autofluorescence removal, multiplexing, and automated analysis methods for in-vivo fluorescence imaging. J. Biomed. Opt..

[85] Weissleder R. (2001). A clearer vision for in vivo imaging. Nat. Biotechnol..

[86] Hoffmann S., Caysa H., Kuntsche J., Kreideweiss P., Leimert A., Mueller T., Mäder K. (2013). Carbohydrate plasma expanders for passive tumor targeting: In vitro and in vivo studies. Carbohyd. Polym..

[87] Han Y.-H., Kankala R.K., Wang S.-B., Chen A.-Z. (2018). Leveraging Engineering of Indocyanine Green-Encapsulated Polymeric Nanocomposites for Biomedical Applications. Nanomaterials.

[88] Kolitz-Domb M., Grinberg I., Corem-Salkmon E., Margel S. (2014). Engineering of near infrared fluorescent proteinoid-poly(L-lactic acid) particles for in vivo colon cancer detection. J. Nanobiotechnol..

[89] Hirsjarvi S., Sancey L., Dufort S., Belloche C., Vanpouille-Box C., Garcion E., Coll J.L., Hindre F., Benoit J.P. (2013). Effect of particle size on the biodistribution of lipid nanocapsules: Comparison between nuclear and fluorescence imaging and counting. Int. J. Pharm..

[90] Studenovský M., Heinrich A.-K., Lucas H., Mueller T., Mäder K., Etrych T. (2016). Dual fluorescent *N*-(2-hydroxypropyl)methacrylamide-based conjugates for passive tumor targeting with reduction-sensitive drug release: Proof of the concept, tumor accumulation, and biodistribution. J. Bioact. Compat. Pol..

[91] Pola R., Heinrich A.K., Mueller T., Kostka L., Mäder K., Pechar M., Etrych T. (2016). Passive Tumor Targeting of Polymer Therapeutics: In Vivo Imaging of Both the Polymer Carrier and the Enzymatically Cleavable Drug Model. Macromol. Biosci..

[92] Cho H., Kwon G.S. (2011). Polymeric Micelles for Neoadjuvant Cancer Therapy and Tumor-Primed Optical Imaging. ACS Nano.

[93] Pola R., Parnica J., Zuska K., Böhmová E., Filipová M., Pechar M., Pankrác J., Mucksová J., Kalina J., Trefil P. (2019). Oligopeptide-targeted polymer nanoprobes for fluorescence-guided endoscopic surgery. Multifunct. Mater..

[94] Ko J.Y., Park S., Lee H., Koo H., Kim M.S., Choi K., Kwon I.C., Jeong S.Y., Kim K., Lee D.S. (2010). pH-Sensitive Nanoflash for Tumoral Acidic pH Imaging in Live Animals. Small.

[95] Gao G.H., Li Y., Lee D.S. (2013). Environmental pH-sensitive polymeric micelles for cancer diagnosis and targeted therapy. J. Control. release.

[96] Etrych T., Daumová L., Pokorná E., Tušková D., Lidický O., Kolářová V., Pankrác J., Šefc L., Chytil P., Klener P. (2018). Effective doxorubicin-based nano-therapeutics for simultaneous malignant lymphoma treatment and lymphoma growth imaging. J. Control. Release.

[97] Berg K., Selbo P.K., Weyergang A., Dietze A., Prasmickaite L., Bonsted A., Engesaeter B.O., Angell-Petersen E., Warloe T., Frandsen N. (2005). Porphyrin-related photosensitizers for cancer imaging and therapeutic applications. J. Microsc-Oxford.

[98] Nakamura H., Liao L., Hitaka Y., Tsukigawa K., Šubr V., Fang J., Ulbrich K., Maeda H. (2013). Micelles of zinc protoporphyrin conjugated to N-(2-hydroxypropyl)methacrylamide (HPMA) copolymer for imaging and light-induced antitumor effects in vivo. J. Control. Release.

[99] Hackbarth S., Islam W., Fang J., Šubr V., Röder B., Etrych T., Maeda H. (2019). Singlet oxygen phosphorescence detection in vivo identifies PDT-induced anoxia in solid tumors. Photochem. Photobiol. Sci..

[100] Niedre M.J., de Kleine R.H., Aikawa E., Kirsch D.G., Weissleder R., Ntziachristos V. (2008). Early photon tomography allows fluorescence detection of lung carcinomas and disease progression in mice in vivo. Proc. Natl. Acad. Sci. USA.

[101] Hall D., Ma G.B., Lesage F., Yong W. (2004). Simple time-domain optical method for estimating the depth and concentration of a fluorescent inclusion in a turbid medium. Opt. Lett..

[102] Swartling J., Svensson J., Bengtsson D., Terike K., Andersson-Engels S. (2005). Fluorescence spectra provide information on the depth of fluorescent lesions in tissue. Appl. Opt..

[103] Shi J.W., Liu F., Pu H.S., Zuo S.M., Luo J.W., Bai J. (2014). An adaptive support driven reweighted L1-regularization algorithm for fluorescence molecular tomography. Biomed. Opt. Express.

[104] Favicchio R., Psycharakis S., Schonig K., Bartsch D., Mamalaki C., Papamatheakis J., Ripoll J., Zacharakis G. (2016). Quantitative performance characterization of three-dimensional noncontact fluorescence molecular tomography. J. Biomed. Opt..

[105] Pian Q., Yao R.Y., Zhao L.L., Intes X. (2015). Hyperspectral time-resolved wide-field fluorescence molecular tomography based on structured light and single-pixel detection. Opt. Lett..

[106] An Y., Liu J., Zhang G.L., Ye J.Z., Du Y., Mao Y., Chi C.W., Tian J. (2015). A Novel Region Reconstruction Method for Fluorescence Molecular Tomography. IEEE Trans. Biomed. Eng..

[107] Chi C., Du Y., Ye J., Kou D., Qiu J., Wang J., Tian J., Chen X. (2014). Intraoperative Imaging-Guided Cancer Surgery: From Current Fluorescence Molecular Imaging Methods to Future Multi-Modality Imaging Technology. Theranostics.

[108] Kelly K., Alencar H., Funovics M., Mahmood U., Weissleder R. (2004). Detection of invasive colon cancer using a novel, targeted, library-derived fluorescent peptide. Cancer Res..

[109] Heffer E., Pera V., Schutz O., Siebold H., Fantini S. (2004). Near-infrared imaging of the human breast: Complementing hemoglobin concentration maps with oxygenation images. J. Biomed. Opt..

[110] Choe R., Corlu A., Lee K., Durduran T., Konecky S.D., Grosicka-Koptyra M., Arridge S.R., Czerniecki B.J., Fraker D.L., DeMichele A. (2005). Diffuse optical tomography of breast cancer during neoadjuvant chemotherapy: A case study with comparison to MRI. Medical. Phys..

[111] Taroni P., Danesini G., Torricelli A., Pifferi A., Spinelli L., Cubeddu R. (2004). Clinical trial of time-resolved scanning optical mammography at 4 wavelengths between 683 and 975 nm. J. Biomed. Opt..

[112] Corlu A., Choe R., Durduran T., Rosen M.A., Schweiger M., Arridge S.R., Schnall M.D., Yodh A.G. (2007). Three-dimensional in vivo fluorescence diffuse optical tomography of breast cancer in humans. Opt. Express.

[113] Intes X., Ripoll J., Chen Y., Nioka S., Yodh A.G., Chance B. (2003). In vivo continuous-wave optical breast imaging enhanced with Indocyanine Green. Med. Phys..

[114] Graves E.E., Ripoll J., Weissleder R., Ntziachristos V. (2003). A submillimeter resolution fluorescence molecular imaging system for small animal imaging. Med. Phys..

[115] Ntziachristos V., Weissleder R. (2001). Experimental three-dimensional fluorescence reconstruction of diffuse media by use of a normalized Born approximation. Opt. Lett..

[116] Ale A., Ermolayev V., Herzog E., Cohrs C., de Angelis M.H., Ntziachristos V. (2012). FMT-XCT: In vivo animal studies with hybrid fluorescence molecular tomography-X-ray computed tomography. Nat. Methods.

[117] Panizzi P., Nahrendorf M., Figueiredo J.L., Panizzi J., Marinelli B., Iwamoto Y., Keliher E., Maddur A.A., Waterman P., Kroh H.K. (2011). In vivo detection of Staphylococcus aureus endocarditis by targeting pathogen-specific prothrombin activation. Nat. Med..

[118] Vonwil D., Christensen J., Fischer S., Ronneberger O., Shastri V.P. (2014). Validation of Fluorescence Molecular Tomography/Micro-CT Multimodal Imaging In Vivo in Rats. Mol. Imaging Biol..

[119] Schulz R.B., Ale A., Sarantopoulos A., Freyer M., Soehngen E., Zientkowska M., Ntziachristos V. (2010). Hybrid System for Simultaneous Fluorescence and X-Ray Computed Tomography. IEEE Trans. Med. Imaging.

[120] Nahrendorf M., Keliher E., Marinelli B., Waterman P., Feruglio P.F., Fexon L., Pivovarov M., Swirski F.K., Pittet M.J., Vinegoni C. (2010). Hybrid PET-optical imaging using targeted probes. Proc. Natl. Acad. Sci. USA.

[121] Ma X., Phi Van V., Kimm M.A., Prakash J., Kessler H., Kosanke K., Feuchtinger A., Aichler M., Gupta A., Rummeny E.J. (2017). Integrin-Targeted Hybrid Fluorescence Molecular Tomography/X-ray Computed Tomography for Imaging Tumor Progression and Early Response in Non-Small Cell Lung Cancer. Neoplasia.

[122] Kunjachan S., Gremse F., Theek B., Koczera P., Pola R., Pechar M., Etrych T., Ulbrich K., Storm G., Kiessling F. (2013). Noninvasive Optical Imaging of Nanomedicine Biodistribution. ACS Nano.

[123] Kunjachan S., Pola R., Gremse F., Theek B., Ehling J., Moeckel D., Hermanns-Sachweh B., Pechar M., Ulbrich K., Hennink W.E. (2014). Passive versus Active Tumor Targeting Using RGD- and NGR-Modified Polymeric Nanomedicines. Nano. Lett..

[124] Giddabasappa A., Gupta V.R., Norberg R., Gupta P., Spilker M.E., Wentland J., Rago B., Eswaraka J., Leal M., Sapra P. (2016). Biodistribution and Targeting of Anti-5T4 Antibody-Drug Conjugate Using Fluorescence Molecular Tomography. Mol. Cancer Ther..

[125] Theek B., Baues M., Gremse F., Pola R., Pechar M., Negwer I., Koynov K., Weber B., Barz M., Jahnen-Dechent W. (2018). Histidine-rich glycoprotein-induced vascular normalization improves EPR-mediated drug targeting to and into tumors. J. Control. Release.

[126] Lee H., Lytton-Jean A.K.R., Chen Y., Love K.T., Park A.I., Karagiannis E.D., Sehgal A., Querbes W., Zurenko C.S., Jayaraman M. (2012). Molecularly self-assembled nucleic acid nanoparticles for targeted in vivo siRNA delivery. Nat. Nanotechnol..

[127] Novobrantseva T.I., Borodovsky A., Wong J., Klebanov B., Zafari M., Yucius K., Querbes W., Ge P., Ruda V.M., Milstein S. (2012). Systemic RNAi-mediated Gene Silencing in Nonhuman Primate and Rodent Myeloid Cells. Mol. Ther-Nucl Acids.

[128] Al Rawashdeh W., Zuo S., Melle A., Appold L., Koletnik S., Tsvetkova Y., Beztsinna N., Pich A., Lammers T., Kiessling F. (2017). Noninvasive Assessment of Elimination and Retention using CT-FMT and Kinetic Whole-body Modeling. Theranostics.

[129] Li B.Q., Maafi F., Berti R., Pouliot P., Rheaume E., Tardif J.C., Lesage F. (2014). Hybrid FMT-MRI applied to in vivo atherosclerosis imaging. Biomed. Opt. Express.

[130] Sosnovik D.E., Nahrendorf M., Deliolanis N., Novikov M., Aikawa E., Josephson L., Rosenzweig A., Weissleder R., Ntziachristos V. (2007). Fluorescence tomography and magnetic resonance imaging of myocardial macrophage infiltration in infarcted myocardium in vivo. Circulation.

[131] Zhang Y., Zhang B., Liu F., Luo J.W., Bai J. (2014). In vivo tomographic imaging with fluorescence and MRI using tumor-targeted dual-labeled nanoparticles. Int. J. Nanomed..

[132] Gaedicke S., Braun F., Prasad S., Machein M., Firat E., Hettich M., Gudihal R., Zhu X., Klingner K., Schüler J. (2014). Noninvasive positron emission tomography and fluorescence imaging of CD133+ tumor stem cells. Proc. Natl. Acad. Sci. USA.

[133] Boutet J., Herve L., Debourdeau M., Guyon L., Peltie P., Dinten J.M., Saroul L., Duboeuf F., Vray D. (2009). Bimodal ultrasound and fluorescence approach for prostate cancer diagnosis. J. Biomed. Opt..

[134] Laidevant A., Herve L., Debourdeau M., Boutet J., Grenier N., Dinten J.M. (2011). Fluorescence time-resolved imaging system embedded in an ultrasound prostate probe. Biomed. Opt. Express.

[135] Theek B., Gremse F., Kunjachan S., Fokong S., Pola R., Pechar M., Deckers R., Storm G., Ehling J., Kiessling F. (2014). Characterizing EPR-mediated passive drug targeting using contrast-enhanced functional ultrasound imaging. J. Control. Release.

[136] McCann C.M., Waterman P., Figueiredo J.L., Aikawa E., Weissleder R., Chen J.W. (2009). Combined magnetic resonance and fluorescence imaging of the living mouse brain reveals glioma response to chemotherapy. Neuroimage.

[137] Penet M.F., Mikhaylova M., Li C., Krishnamachary B., Glunde K., Pathak A.P., Bhujwalla Z.M. (2010). Applications of molecular MRI and optical imaging in cancer. Future Med. Chem..

[138] Mikhaylova M., Stasinopoulos I., Kato Y., Artemov D., Bhujwalla Z.M. (2009). Imaging of cationic multifunctional liposome-mediated delivery of COX-2 siRNA. Cancer Gene Ther..

[139] Medarova Z., Pham W., Farrar C., Petkova V., Moore A. (2007). In vivo imaging of siRNA delivery and silencing in tumors. Nat. Med..

